# A comprehensive database of the geographic spread of past human Ebola outbreaks

**DOI:** 10.1038/sdata.2014.42

**Published:** 2014-10-23

**Authors:** Adrian Mylne, Oliver J. Brady, Zhi Huang, David M. Pigott, Nick Golding, Moritz U.G. Kraemer, Simon I. Hay

**Affiliations:** 1 Spatial Ecology & Epidemiology Group, Department of Zoology, University of Oxford, Oxford, OX1 3PS, UK; 2 Fogarty International Center, National Institutes of Health, Bethesda, MD 20892-2220, USA.

## Abstract

Ebola is a zoonotic filovirus that has the potential to cause outbreaks of variable magnitude in human populations. This database collates our existing knowledge of all known human outbreaks of Ebola for the first time by extracting details of their suspected zoonotic origin and subsequent human-to-human spread from a range of published and non-published sources. In total, 22 unique Ebola outbreaks were identified, composed of 117 unique geographic transmission clusters. Details of the index case and geographic spread of secondary and imported cases were recorded as well as summaries of patient numbers and case fatality rates. A brief text summary describing suspected routes and means of spread for each outbreak was also included. While we cannot yet include the ongoing Guinea and DRC outbreaks until they are over, these data and compiled maps can be used to gain an improved understanding of the initial spread of past Ebola outbreaks and help evaluate surveillance and control guidelines for limiting the spread of future epidemics.

## Background & Summary

**Figure 1:**
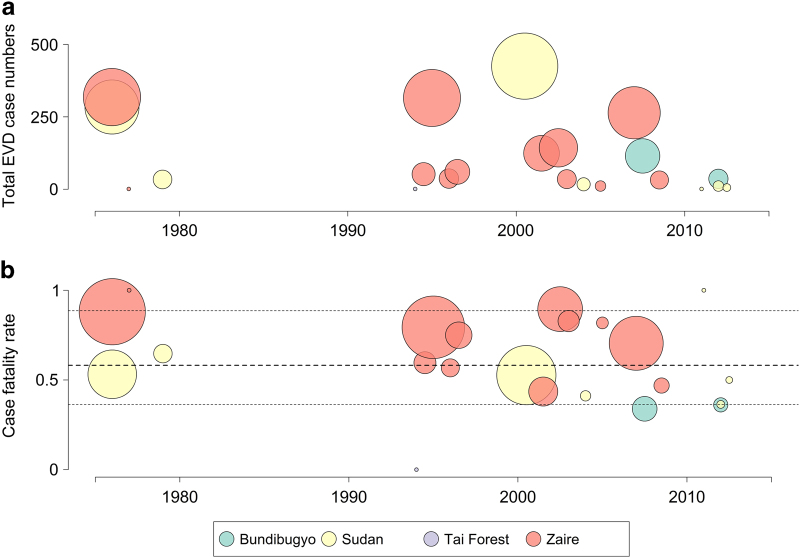
The size (**a**) and case fatality rate (**b**) of the 22 previous Ebola outbreaks (suspected and confirmed cases). Circle area is proportional to the total number of reported cases (**a**) or deaths (**b**) for each outbreak. Circle colour represents different species of Ebolavirus. Black dotted lines in (**b**) show the median and upper and lower 75% quantiles of outbreak case fatality rate.

The genus *Ebolavirus* belongs to the family *Filoviridae* and contains five known species to date that vary in their distribution, reservoir hosts and their pathogenicity to humans. With the exception of *Reston ebolavirus* which has only shown pathogenicity among primates and porcids, all of these have shown some capacity to spill over from their natural reservoirs and cause human cases^1,2^. While only a single human case of *Tai Forest ebolavirus* has been documented^3^, the remaining three species (*Zaire ebolavirus*, *Sudan ebolavirus* and *Bundibugyo ebolavirus*) are all known to permit human to human transmission after the initial suspected zoonotic transfer resulting in outbreaks of different sizes, geographic extents and case fatality rates (Fig. 1)^4,5^.

Which reservoir species are responsible for maintaining Ebolavirus transmission between outbreaks is not well understood^6^, but several candidate species have been identified. In Gabon three species of bats (*Hypsignathus monstrosus*, *Epomops franqueti* and *Myonycteris torquata*) were found to be infected with Ebola virus^7^ and some human outbreaks have been directly linked to bat exposure^8^. While it is increasingly clear that gorillas (*Gorilla gorilla*) and chimpanzees (*Pan troglodytes*) are dead end hosts for the virus, infection in populations of these species is frequently found and they still present a risk of animal to human transmission^3,9–14^. For many outbreaks it has been difficult to definitively identify the source of human Ebola index cases, but activities that bring humans into close contact with the blood of mammals through activities such as hunting and the bushmeat trade are common to many of the index cases^8,14–16^.

Following the initial suspected zoonotic transfer, secondary transmission can result from close contact between infectious individuals or corpses and other humans, usually through exposure to infectious bodily fluids^2^. Due to the close degree of contact required for secondary transmission, certain specific community activities are commonly associated with hotspots of secondary transmission such as family home care, traditional burial practices that involve washing the corpse, or healthcare settings where sufficient protective measures are not in place^15,17,18^. These focal transmission events combined with an incubation period of 5–9 days mean transmission can often be observed in waves of cases^2,18^ in both space and time.

Spread of cases over longer distances is often associated with treatment seeking that draws people from rural villages that typify the index case locations to big urban centres with central medical facilities (Supplementary Figures 1–22). While this mostly involves domestic land travel^19^, some instances of international importation by air travel have been documented^18^. Following travel of an infectious individual, either secondary clusters of Ebola cases will occur, or transmission will be interrupted by control methods such as quarantine and patient contact tracing^20,21^. Due to the variable rate of progression of symptoms of Ebola virus disease (EVD) (onset of Ebola Haemorrhagic fever can range from 2–21 days^2^), case fatality rates can vary significantly depending on a number of factors associated with Ebola virus pathogenesis and the quality and timing of symptomatic care. The complex interaction between surveillance, control, treatment seeking, patient-contact rates and their combined effects on the dynamics of transmission dictate the spread, magnitude and case fatality rate of an Ebola outbreak.

This database collates existing knowledge on the geographic spread of past Ebola outbreaks in a standardised format that allows the dynamics of different outbreaks to be compared. Procedures for data abstraction are outlined and each outbreak is summarised with a map and brief text description. These data will be useful for conducting spatial analyses of Ebola outbreak spread. We include every outbreak preceding the atypical 2013 Guinea epidemic which has spread further and faster than any previous epidemic. Once the current Guinea and Democratic Republic of the Congo (DRC) outbreaks are over, this database will be updated to include the same standardised data fields for these contemporary outbreaks. Periodic updates to include any additional Ebola outbreaks will also ensure this resource has on-going relevance in Ebola spread analyses. In particular, a comparison between the Guinea 2013 outbreak and historical outbreaks will allow an evaluation of surveillance and control guidelines in terms of their appropriateness for mitigating the spread of future Ebola outbreaks of variable magnitude. In the meantime, it is hoped that these data will support research into EVD epidemiology which can be brought to bear on the current outbreak.

## Methods

### Data collection

Tables listing proven outbreaks of Ebola virus, sourced from the scientific literature^5^ and from health reporting organisations^22^, were used to coordinate initial searches of the formal scientific literature using Web of Science and PubMed for each specific outbreak. Relevant papers were abstracted and, where possible, outbreak-specific epidemiological surveys were sourced. The citations in these references were obtained in order to reconstruct the outbreak in detail and extract a range of epidemiological data relating to geographic spread, case and fatality numbers.

In this analysis we excluded ongoing outbreaks meaning that the current Guinea and DRC spread data are not yet included. When this outbreak is over and data has been assimilated we will update this database to incorporate the Guinea and DRC outbreaks using the same procedures described below, which will allow a comparison with historical outbreaks of Ebola.

### Definitions of index, secondary and imported cases

Index cases were defined as any human infection resulting from interaction with non-human sources of the disease. Among the sources examined, index cases were identified based on reported interaction with suspected zoonotic reservoirs or hosts, such as non-human mammals during hunting trips^8,14–16,23^. In cases where a mode of suspected zoonotic transmission could not be established, the first reported case was assumed to be the index case. Any cases arising from existing human infections are considered as secondary infections. Cases reported after the index cases were assumed to be secondary cases unless they were accompanied by specific details of likely exposure to a zoonotic reservoir or non-human host. If a case was reported in an area where no further cases occurred and no continued transmission was documented, these were termed imported cases.

### Procedure for geo-positioning

For each index, secondary or imported case cluster that could be linked to a unique geographic location we performed a range of procedures similar to methods employed elsewhere^24,25^ to assign geographic coordinates. For index and secondary cases these locations were representative of the site of suspected zoonotic transfer or human-to-human transmission respectively. Index cases were geopositioned as the location where exposure to the suspected zoonotic reservoir was likely to be highest. For example hunters who butchered carcases on hunting expeditions were geopositioned as a polygon covering the area of the hunting trip not a point at the hunters’ homes. In contrast, if an individual purchased bushmeat from a local market for preparation and consumption at home, the home of the individual was georeferenced as the index case, as it was considered the location of highest exposure to the suspected zoonotic reservoir. For imported cases these locations were representative of all locations that infected patients travelled to, but did not cause onward transmission. For the purpose of this analysis we excluded international imported cases that did not cause autochthonous secondary transmission as, in most cases, they represented diagnosed foreign workers or expatriates who were evacuated for specialised treatment^3,10^. In such circumstances appropriate preventative measures are employed meaning that risk of onward transmission is negligible.

Each occurrence was reported to the highest degree of spatial resolution available based upon the information provided, as long as they could be categorised into one of: index, secondary or imported cases. This ranged from point locations (indicative of a precise location, such as a village), to areas, termed polygon locations, which correspond approximately to administrative regions or custom digitised areas based on site descriptors within the primary articles. Administrative regions were defined as classified by the Food and Agriculture Organization’s Global Administrative Unit Layers (GAUL) coding^26^. These classify national boundaries as admin0 units, states or provinces as admin1 units and districts as admin2 units. By classifying Ebola occurrences as polygons we were able to represent the geographic uncertainty around the exact location of Ebola transmission which could have occurred anywhere within the defined region. For towns, the coordinates of the centre were recorded, unless a specific part of the town (or an explicit latitude and longitude) was described. Coordinates for point locations were extracted using Google Earth (version 7.1.2). If the area concerned could not be assigned to a finer resolution than 5 km×5 km, it was entered as polygon rather than point data. If specified regions could not be linked to an admin2 or admin1 unit, custom polygons were digitised using site descriptions in the text articles. For imprecise descriptors e.g., ‘15 km from the town’ with no direction specified or ‘cases occurred on a north-south road between village X and Y’, circular polygons were digitised based on radius distances given or extreme points that defined the geographic limits of transmission. These circular polygons could be trimmed if their area included admin1 or admin2 administrative regions which reported no EVD patients. Some articles referred to ‘healthcare districts’ that did not correspond to admin1 or admin2 units, but were definable based on maps presented in the primary literature that were digitised or were available on the map sharing website IKI (www.ikimap.com)^27^. For index cases that referred to suspected zoonotic transfer in specific forests or game reserves, polygons were drawn based on the specified park or forest geographic boundary as shown in Google Earth. Two exceptional cases were present. In the first, for outbreak 13, the index case transmission site description merely mentioned a case being reported as ‘near the town of Mbandza’. In this instance a circular polygon was defined with radius of half the distance to the next specified location of transmission (7.5 km)^15^. In the second, for outbreak 9, two locations described in the primary literature could not be located, but were described as ‘near to the town of Booue’^18^. As a result the same procedure was undertaken and a radius of 30 km was defined around the village of Booue. All digitising was performed using ArcGIS 10.1^28^.

### Key outbreak metrics recorded

The total number of cases for each outbreak was obtained from the most recent primary source (Table 1). Cases included both clinically suspected and laboratory confirmed cases at the point of care, or diagnosed retrospectively. The number of people who died with a suspected or confirmed diagnosis of EVD was also recorded. These data were spatially disaggregated as much as possible from the information given in the text to give measures of spatial variation in case fatality rate within an outbreak.

Outbreak start and end time were defined by reports of the first (index) and last cases respectively. For each secondary and imported case that occurred in a novel location, the source of importation was recorded where reported. When possible this was reconciled with the date of the first secondary or imported case in each cluster to determine geographic spread order. We only included confirmed sources of origin, as determined by detailed patient histories, not suspected sources and no differentiation of spread order to two new clusters from the same source was made unless both new clusters documented the date of their first secondary case. If two secondary case clusters had a confirmed source but no differentiation of order we assigned them the same spread order. Similarly if a secondary case cluster could have come from two specified sources both were included as sources of spread. Due to a lack of epidemiological investigation at the time, or through information being lost in the reporting chain, the spread order of many secondary and imported case clusters was partially complete or missing altogether (Table 2).

For each outbreak the above details were combined with additional information from the original articles on method and timing of spread to construct the text descriptions accompanying the spread maps in Supplementary Figures 1–22 (selected examples in Figs. 2,3,4). Spread is defined as movement of an infected individual from the location of infection to a transmission-free area. For example an individual who was likely infected from the suspected zoonotic reservoir on a hunting expedition in the forest then caused secondary infections back in their local village, would qualify as having spread the virus from the forest to the village. By contrast if the first reported case occurred in a village where subsequent secondary cases then occurred, no spread would have been recorded as index and secondary transmission occur in the same location.

**Figure 2:**
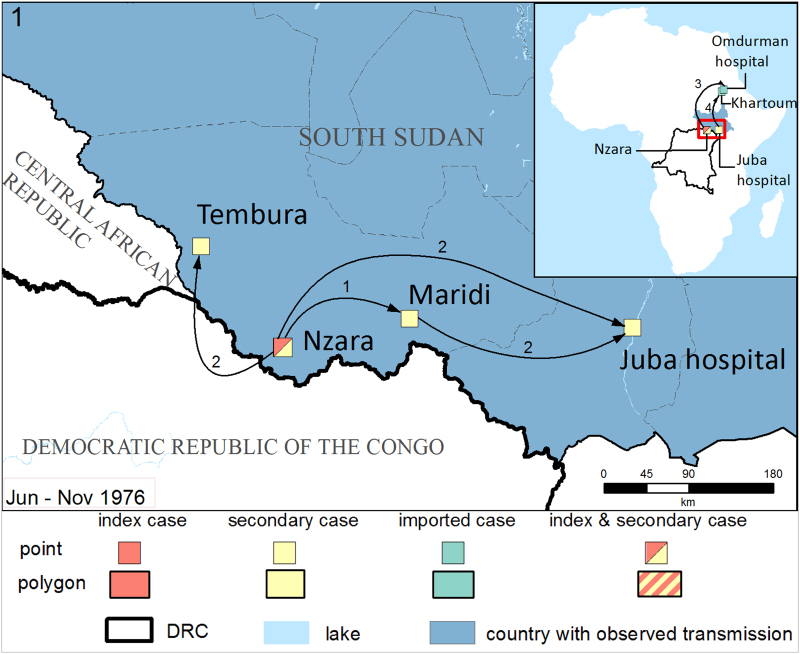
Map of the South Sudan (1976) outbreak. The first reported cases of Sudan Ebola virus were in three workers at a cotton factory in Nzara, in close proximity to three game reserves. The method of acquisition was unknown. The first secondary cases arose in Nzara infecting a total of 67 people who were primarily family members of the factory workers. Further secondary transmission clusters emerged in Maridi following spread from Nzara due to seeking treatment, after which further cases occurred in Juba due to patients who were referred. Additional cases from Maridi were also referred directly to Juba making the source of infection in Juba difficult to identify. Secondary transmission also emerged in Tembura due to a patient seeking family care, although the source of this infection is unknown. Imported cases from Juba to Khartoum and from Nzara to Omdurman were also reported following a patient seeking treatment and a referral for diagnosis respectively (see inset). The principal mode of transmission in this outbreak was initially familial, although in Maridi secondary transmission arose through nosocomial transmission. Seeking of treatment was the principal cause of geographic spread. The first index case became ill on the 27 June 1976 before the first secondary cases in July and subsequent secondary transmission clusters from August to October. Cases peaked in September (138 cases, 65 deaths). The final case was reported on 25 November 1976. Imported cases in Omdurman and Khartoum were reported in August and September, respectively. Overall, 284 cases were reported with 151 deaths giving a CFR of 53.2%. This figure varied in different locations: Nzara (67,31,46%), Maridi (213,116,55%), Tembura (3,3,100%), Juba (1,1,100%). Arrows indicate order of spread. Where spread order is known, numbers are indicative of the order of spread. Arrows sharing the same number indicate that it was not possible to distinguish which spread happened first.

**Figure 3:**
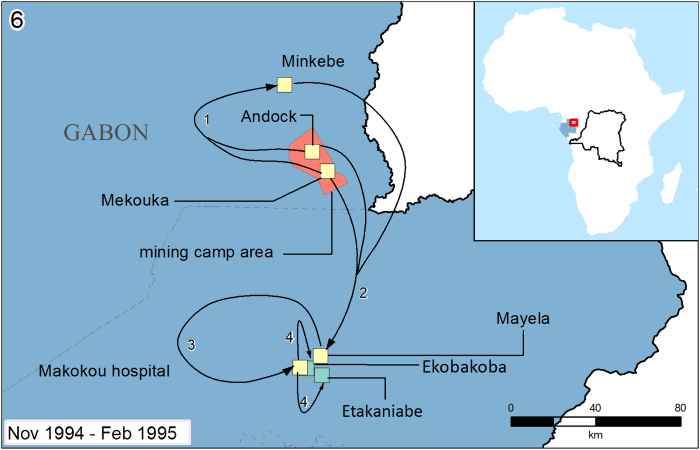
Map of the Gabon (1994) outbreak. The first reported cases of Zaire Ebola virus were in miners from the Mekouka and Andock encampments, suspected to have contracted the infection in the surrounding area. The method of acquisition was unknown. The first secondary cases arose within these two encampments and then spread to the Minkebe camp. Further secondary transmission clusters emerged in Mayela then Makokou general hospital after 32 patients from the forest encampments sought treatment. Cases were also reported in Ekataniabe and Ekobakoba who had recent travel histories to Makokou general hospital. The principal modes of transmission were among workers at first, followed by nosocomial in Makokou general hospital and familial in Mayela (connected by a single traditional healer). The initial case was reported on 13 November 1994 before secondary transmission clusters occurred from the end of January to February 1995. Cases and deaths peaked in December (26 cases, 14 deaths (53.8% CFR)). The final case was reported on 9 February 1995 in Ekobakoba. Overall, 49 cases were reported with 30 deaths, giving a CFR of 61.2%. For map key, see Fig. 2.

**Figure 4:**
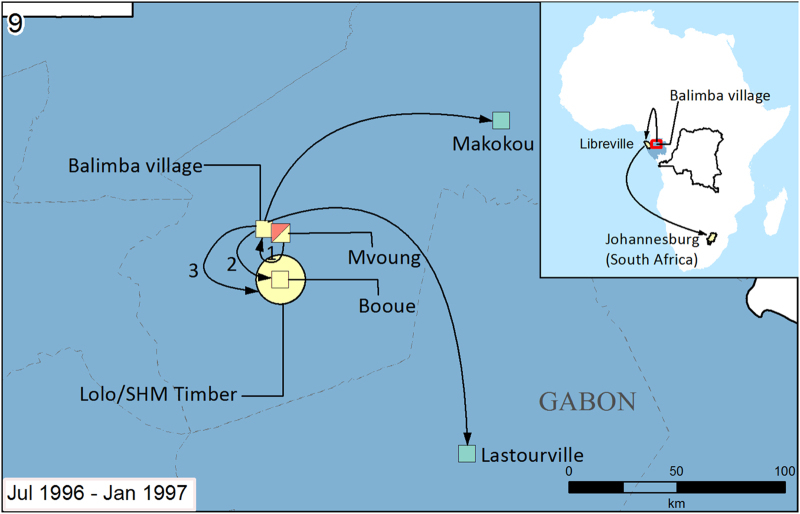
Map of the Gabon (1996b) outbreak. The index case of Zaire Ebola virus likely came from one of three infected hunters in a logging camp near Mvoung. The timing of infection makes it difficult to distinguish index cases from secondary cases during the early stages of this outbreak, but it is likely that the first secondary cases emerged amongst the hunters who then sought treatment from a traditional healer in Balimba. After falling ill, the traditional healer from Balimba sought treatment in Booue, where the disease then radially spread through the communities in the surrounding areas. A further secondary transmission cluster emerged in Libreville (see inset) after patients from Balimba sought treatment there. In Libreville one doctor became infected and flew to Johannesburg, South Africa for treatment before receiving a diagnosis of Ebola. Limited further nosocomial transmission (1 case) occurred upon his arrival in Johannesburg. Imported cases in Makokou General Hospital and Lastourville were also reported after patients from Balimba sought treatment. No clear principal mode of transmission was observed for the early stages of the outbreak, but in Libreville secondary transmission mainly arose through nosocomial transmission. The index case was reported on the 13 July 1976 before the first secondary cases in September and subsequent secondary transmission clusters from September to January. Cases peaked in September and deaths peaked in October. The final case was reported on 18 January 1997. Overall 60 cases were reported with 45 deaths, giving a CFR of 75%. For map key, see Fig. 2.

Selected examples are presented in Figs. 2,3,4. In the first, initial focal infection jumped from village to village, as well as longer distance dispersal primarily through treatment seeking of infected patients (Fig. 2). In the second (Fig. 3) more limited outbreak, cases occurred in two main clusters, with one containing working camps surrounding the site of suspected zoonotic transfer and the other villages and a local hospital in the vicinity of Makokou. In the final example outbreak (Fig. 4) secondary transmission spread radially out from the village of Balimba following treatment seeking of a faith healer. Similar to outbreak 1, infected patients travelled to local and national healthcare centres for treatment, in some cases causing secondary transmission at great distances from the index case.

In the text descriptions, methods, sources and order of spread were described in a standardised manner, where information allowed. A brief summary of the evolution of the outbreak over time and the breakdown of total number of cases, deaths and case fatality rate over the course of the entire outbreak is also given.

A wider-scale map showing the locations of all the outbreaks since 1976 is also available in Supplementary Fig. 23 and a table detailing which type of data were obtained from which source for each outbreak is given in Table 1 ^3,8,10,13–18,20,29–46^.

## Data Records

The data from this analysis are summarised in two types of data format (Data Citation 1). First a data table details unique geographic locations of Ebola occurrence, including information on type of transmission, location, spread, timing and case number. These geographic locations are grouped into individual outbreaks (*n*=22) and summary statistics on timings and case and death numbers are given for each outbreak. Second, geographic information files are provided that match the information presented in the data table to explicit geographic areas. These are available in a variety of formats that can be read by geographic information system (GIS) applications. Information from these two file types were used to make the outbreak summary maps and text in Supplementary Figures 1–22 (selected examples in Figs. 2,3,4).

### Data table of unique Ebola virus transmission locations

The table includes the following fields, detailed below. The value ‘NA’ was entered if information was unknown, unreported or indeterminable. The term ‘occurrence’ refers to Ebola transmission locations that are either unique in the geographic location or in their type of transmission (index, secondary or imported). Each row in the table represents a unique Ebola occurrence. A group of occurrences make up contained ‘outbreaks’ and fields with the prefix ‘OB’ summarise various metrics related to the entire outbreak that each occurrence belongs to.

**UNIQ_ID**: A unique identification number for each occurrence at which index, secondary or imported cases of EVD have occurred at unique geographic locations (*n*=117).

**NAME**: Text description of the point or polygon that defines the location of the occurrence.

**COUNTRY:** The country where the majority of cases occurred in each outbreak.

**VIRUS:** The Ebola virus species of each outbreak.

**CASE_TYPE:** The type of transmission represented by the Ebola occurrence. Can be either ‘index’, ‘secondary’ or ‘import’.

**DATA_TYPE:** Whether the occurrence represents a point or larger polygon location.

**LAT:** The latitude of the centre point of the point or polygon of the occurrence.

**LONG:** The longitude of the centre point of the point or polygon of the occurrence.

**LOC_NTS:** Additional notes describing the site location of the occurrence.

**SPR_ORDER:** The order of spread between occurrences over the course of the outbreak, as determined by the date of onset of the first case in a given occurrence. Index cases are represented with the value ‘1’. Two or more occurrences share the same spread order if it is unknown which of the two areas Ebola virus transmission spread to first.

**SOURCE_1:** The unique identification number of the occurrence where the first EVD patient came from.

**SOURCE_2:** The unique identification number of the occurrence where the first EVD patient came from. An occurrence may have more than one source if infected patients came from more than one source but it is unknown which triggered secondary transmission.

**SOURCE_3:** The unique identification number of the occurrence where the first EVD patient came from. An occurrence may have more than one source if infected patients came from more than one source but it is unknown which triggered secondary transmission.

**STR_DAY:** Day of first reported case in the occurrence.

**STR_MNTH:** Month of first reported case in the occurrence.

**STR_YEAR:** Year of first reported case in the occurrence.

**END_DAY:** Day of last reported case in the occurrence.

**END_MNTH:** Month of last reported case in the occurrence.

**END_YEAR:** Year of last reported case in the occurrence.

**REP_CASE:** The total number of cases (suspected or confirmed) reported over the course of the outbreak, but only within the occurrence.

**REP_DEATH:** The total number of deaths (suspected or confirmed) reported over the course of the outbreak, but only within the occurrence.

**OB_ID:** A unique identification number for each outbreak (*n*=22).

**OB_STR_DAY:** Day of first reported case of the outbreak.

**OB_STR_MNTH:** Month of first reported case of the outbreak.

**OB_STR_YEAR:** Year of first reported case of the outbreak.

**OB_END_DAY:** Day of last reported case of the outbreak.

**OB_END_MNTH:** Month of last reported case of the outbreak.

**OB_END_YEAR:** Year of last reported case of the outbreak.

**OB_CASE:** The total number of cases (suspected or confirmed) reported over the course of the outbreak in all areas.

**OB_DEATH:** The total number of deaths (suspected or confirmed) reported over the course of the outbreak in all areas.

### Geographic information files

The geographic information files include index, secondary and imported cases linked to geographic locations. All fields match those in the data table. Unknown, unreported or indeterminable data are represented by the character combination ‘NA’.

## Technical Validation

For each outbreak, all relevant text articles were used to confirm or reach a consensus on the likely result. Where differing results were found (e.g., total case count), figures from primary research articles took preference over review, summary articles or periodical epidemiological reports.

The extracted geographic and epidemiological summary information was cross-checked by at least two different researchers to ensure accuracy.

All point and polygon locations were checked in ArcGIS 10.1^28^ against national and subnational boundaries to ensure their location matched the text descriptions. A gridded raster file from the Global Lakes and Wetlands Database^47^ giving the locations of rivers and lakes was also included to check points and polygons fell on land rather than water. Any points that fell in water were moved to the nearest land pixel.

## Usage Notes

The data presented here can be used in combination with spatial and temporal meteorological and socioeconomic information to generate hypotheses about the factors that may be important in the emergence of index cases and the spread of secondary and imported cases of EVD.

Pigott *et al.* combined data on Ebola index cases with a suite of environmental information in a species distribution model to map the zoonotic niche of Ebola transmission across Africa^48^. Matching more varied and finer scale local information at the sites of the index cases presented here could help develop our understanding of the complex process of Ebola virus emergence and the risk posed by certain human activities and land use patterns.

Using the secondary and imported case data, it would be possible to model and investigate causes of spread of human Ebola outbreaks. Understanding spread of the pathogen in past outbreaks may aid control of the current outbreak. A comparison between the spread of historical outbreaks and the spread of the current ongoing outbreak once it is over will be useful for informing Ebola surveillance and control guidelines to minimise the size and burden of these sporadic zoonoses.

Finally, this dataset can be used to investigate how the rate, extent and the environment in which Ebola outbreaks spread relates to important outbreak measures such as the total number of cases and case fatality rate. Understanding what distinguishes brief, geographically limited and low mortality Ebola outbreaks from those that impose a much higher public health burden will be important for informing future surveillance, control and treatment efforts^49^. This dataset provides the most comprehensive collection of standardised data on Ebola outbreak spread currently available and will be an important resource for these uses. These records will also be updated periodically with data from ongoing and future Ebola outbreaks to ensure the ongoing relevance of this database.

## Additional information

**How to cite this article:** Mylne, A. *et al.* A comprehensive database of the geographic spread of past human Ebola outbreaks. *Sci. Data* 1:140042 doi: 10.1038/sdata.2014.42 (2014).

## Supplementary Material

Supplementary Figures

## Figures and Tables

**Table 1:** Types of information extracted from each information source.

Outbreak	Source	Type of information available
1. Sudan 1976	WHO/International study team. Ebola haemorrhagic fever in Sudan, 1976. *Bull. World Health Organ.* **56**, 247-270 (1978).	Case numbers (284), fatality numbers (151), dates and spread locations
2. DRC 1976	International Commission. Ebola haemorrhagic fever in Zaire, 1976. *Bull. World Health Organ.* **56**, 271-293 (1978).	Case numbers (318), fatality numbers (280), dates and spread locations
3. DRC 1977	Heymann, D. *et al.* Ebola hemorrhagic fever: Tandala, Zaire, 1977–1978. *J. Infect. Dis.* **142**, 372-376 (1980).	Case numbers (1), fatality numbers (1), dates and spread locations
4. South Sudan 1979	Baron R. C., McCormick, J. B. & Zubeir, O. A. Ebola virus disease in southern Sudan - hospital dissemination and intrafamilial spread. *Bull. World Health Organ.* **61**, 997-1003 (1983).	Case numbers (34), fatality numbers (22), dates and spread locations
5. Côte d’Ivoire 1994	Le Guenno, B. *et al.* Isolation and partial characterisation of a new strain of Ebola virus. *The Lancet* **345**, 1271-1274 (1995).	Case numbers (1), fatality numbers (0) and dates
	Formenty, P. *et al.* Ebola virus outbreak among wild chimpanzees living in a rain forest of Cote d'Ivoire. *J. Infect. Dis.* **179**, S120-S126 (1999).	Spread locations
6. Gabon 1994	Georges, A.-J. *et al.* Ebola hemorrhagic fever outbreaks in Gabon, 1994–1997: epidemiologic and health control issues. *J. Infect. Dis.* **179**, S65-S75 (1999).	Case numbers (49)*, fatality numbers (30)*, dates and spread locations
	Amblard, J. *et al.* Identification of the Ebola virus in Gabon in 1994. *The Lancet* **349**, 181-182 (1997).	Case numbers (44), fatality numbers (28) and spread locations
	Milleliri, J., Tévi-Benissan, C., Baize, S., Leroy, E. & Georges-Courbot, M. Les épidémies de fièvre hémorragique due au virus Ebola au Gabon (1994-2002). *Bull. Soc. Pathol. Exot. Filiales* **97**, 199-205 (2004).	Case numbers (51), fatality numbers (31) and spread locations
7. DRC 1995	Khan, A. S. *et al.* The reemergence of Ebola hemorrhagic fever, Democratic Republic of the Congo, 1995. *J. Infect. Dis.* **179**, S76-S86 (1999).	Case numbers (315), fatality numbers (250), dates and spread locations
	Muyembe, T. & Kipasa, M. Ebola haemorrhagic fever in Kikwit, Zaire. *The Lancet* **345**, 1448 (1995).	Spread locations
8. Gabon 1996	Georges, A.-J. *et al.* Ebola hemorrhagic fever outbreaks in Gabon, 1994–1997: epidemiologic and health control issues. *J. Infect. Dis.* **179**, S65-S75 (1999).	Case numbers (31), fatality numbers (21), dates and spread locations
	Milleliri, J., Tévi-Benissan, C., Baize, S., Leroy, E. & Georges-Courbot, M. Les épidémies de fièvre hémorragique due au virus Ebola au Gabon (1994-2002). *Bull. Soc. Pathol. Exot. Filiales* **97**, 199-205 (2004).	Case numbers (31), fatality numbers (21) and spread locations
9. Gabon 1996b	Georges, A.-J. *et al.* Ebola hemorrhagic fever outbreaks in Gabon, 1994–1997: epidemiologic and health control issues. *J. Infect. Dis.* **179**, S65-S75 (1999).	Case numbers (60), fatality numbers (45), dates and spread locations
	Milleliri, J., Tévi-Benissan, C., Baize, S., Leroy, E. & Georges-Courbot, M. Les épidémies de fièvre hémorragique due au virus Ebola au Gabon (1994-2002). *Bull. Soc. Pathol. Exot. Filiales* **97**, 199-205 (2004).	Case numbers (60), fatality numbers (45) and spread locations
10. Uganda 2000	Lamunu, M. *et al.* Containing a haemorrhagic fever epidemic: the Ebola experience in Uganda (October 2000–January 2001). *Int. J. Infect. Dis.* **8**, 27-37 (2004).	Case numbers (60), fatality numbers (45), dates and spread locations
	World Health Organization. Outbreak of Ebola haemorrhagic fever, Uganda, August 2000–January 2001. *Wkly. Epidemiol. Rec.* **76**, 41-48 (2001).	Case numbers (60), fatality numbers (45), dates and spread locations
	Okware, S. *et al.* An outbreak of Ebola in Uganda. *Trop. Med. Int. Health* **7**, 1068-1075 (2002).	Case numbers (60), fatality numbers (45), dates and spread locations
11. Gabon 2001	Nkoghe Mba, D. *et al.* Plusieurs épidémies de fièvre hémorragique due au virus Ebola au Gabon, d'octobre 2001 à avril 2002. *Bull. Soc. Pathol. Exot. Filiales* **98**, 224-229 (2005).	Case numbers (124)*, fatality numbers (97)*, dates and spread locations
	World Health Organization. Outbreak(s) of Ebola haemorrhagic fever in the Republic of the Congo, January-April 2003. *Wkly. Epidemiol. Rec.* **78**, 285-289 (2003).	Case numbers (97), fatality numbers (73), dates and spread locations
	Milleliri, J., Tévi-Benissan, C., Baize, S., Leroy, E. & Georges-Courbot, M. Les épidémies de fièvre hémorragique due au virus Ebola au Gabon (1994-2002). *Bull. Soc. Pathol. Exot. Filiales* **97**, 199-205 (2004).	Case numbers (124)*, fatality numbers (97)* and spread locations
12. Gabon 2001b	World Health Organization. Outbreak(s) of Ebola haemorrhagic fever in the Republic of the Congo, January-April 2003. *Wkly. Epidemiol. Rec.* **78**, 285-289 (2003).	Case numbers (143), fatality numbers (128), dates and spread locations
13. RoC 2003	Boumandouki, P. *et al.* Prise en charge des malades et des défunts lors de l'épidémie de fièvre hémorragique due au virus Ebola d’octobre à décembre 2003 au Congo. *Bull. Soc. Pathol. Exot. Filiales* **98**, 218-223 (2005).	Case numbers (35), fatality numbers (29), dates and spread locations
14. South Sudan 2004	World Health Organization. Outbreak of Ebola haemorrhagic fever in Yambio, south Sudan, April-June 2004. *Wkly. Epidemiol. Rec.* **80**, 370-375 (2005).	Case numbers (17), fatality numbers (7), dates and spread locations
	Onyango, C. O. *et al.* Laboratory diagnosis of Ebola hemorrhagic fever during an outbreak in Yambio, Sudan, 2004. *J. Infect. Dis.* **196**, S193-S198 (2007).	Spread locations
15. RoC 2005	Nkoghe, D., Kone, M. L., Yada, A. & Leroy, E. A limited outbreak of Ebola haemorrhagic fever in Etoumbi, Republic of Congo, 2005. *Trans. R. Soc. Trop. Med. Hyg.* **105**, 466-472 (2011).	Case numbers (12), fatality numbers (10), dates and spread locations
16. DRC 2007	Leroy, E. M. *et al.* Human Ebola outbreak resulting from direct exposure to fruit bats in Luebo, Democratic Republic of Congo, 2007. *Vector Borne Zoonot. Dis.* **9**, 723-728 (2009).	Case numbers (264), fatality numbers (186), dates and spread locations
	Grard, G. *et al.* Emergence of divergent Zaire ebola virus strains in Democratic Republic of the Congo in 2007 and 2008. *J. Infect. Dis.* **204**, S776-S784 (2011).	Spread locations
17. Uganda 2007	Wamala, J. F. *et al.* Ebola hemorrhagic fever associated with novel virus strain, Uganda, 2007–2008. *Emerg. Infect. Dis.* **16**, 1087-1092 (2010).	Case numbers (116), fatality numbers (39), dates and spread locations
	MacNeil, A. *et al.* Proportion of deaths and clinical features in Bundibugyo Ebola virus infection, Uganda. *Emerg. Infect. Dis.* **16**, 1969 (2010).	Spread locations
	Towner, J. S. *et al.* Newly discovered ebola virus associated with hemorrhagic fever outbreak in Uganda. *PLoS Pathog.* **4**, e1000212 (2008).	Spread locations
18. DRC 2008	World Health Organisation. *End of Ebola outbreak in the Democratic Republic of the Congo* http://www.who.int/csr/don/2009_02_17/en/ (2009).	Case numbers (32)*, fatality numbers (15)*, dates and spread locations
	Grard, G. *et al.* Emergence of divergent Zaire ebola virus strains in Democratic Republic of the Congo in 2007 and 2008. *J. Infect. Dis.* **204**, S776-S784 (2011).	Case numbers (14), fatality numbers (10), dates and spread locations
19. Uganda 2011	Shoemaker, T. *et al.* Reemerging Sudan ebola virus disease in Uganda, 2011. *Emerg. Infect. Dis.* **18**, 1480 (2012).	Case numbers (1), fatality numbers (1), dates and spread locations
20. DRC 2012	Albarino, C. *et al.* Genomic analysis of filoviruses associated with four viral hemorrhagic fever outbreaks in Uganda and the Democratic Republic of the Congo in 2012. *Virology* **442**, 97-100 (2013).	Case numbers (36)*, fatality numbers (13)*, dates and spread locations
	World Health Organization. *DR Congo: Ebola (Situation as of 01 October 2012)* http://www.afro.who.int/pt/grupos-organicos-e-programas/ddc/alerta-e-resposta-epidemias-e-pandemias/outbreak-news/3698-dr-congo-ebola-situation-as-of-01-october-2012.html (2012).	Case numbers (31), fatality numbers (10) and spread locations
21. Uganda 2012	Albarino, C. *et al.* Genomic analysis of filoviruses associated with four viral hemorrhagic fever outbreaks in Uganda and the Democratic Republic of the Congo in 2012. *Virology* **442**, 97-100 (2013).	Case numbers (11)*, fatality numbers (4)*, dates and spread locations
	World Health Organisation. *Ebola in Uganda* http://www.who.int/csr/don/2012_07_29/en/ (2012).	Case numbers (20), fatality numbers (14) and spread locations
	World Health Organisation. *Uganda : Ebola (situation as of 27 August 2012)* http://www.afro.who.int/en/clusters-a-programmes/dpc/epidemic-a-pandemic-alert-and-response/outbreak-news/3674-uganda--ebola-situation-as-of-27-august-2012-.html (2012).	Case numbers (24), fatality numbers (17) and spread locations
22. Uganda 2012b	Albarino, C. *et al.* Genomic analysis of filoviruses associated with four viral hemorrhagic fever outbreaks in Uganda and the Democratic Republic of the Congo in 2012. *Virology* **442**, 97-100 (2013).	Case numbers (6), fatality numbers (3), dates and spread locations
Where two different sources disagreed on data the latest primary source was chosen, as indicated by a *. DRC = Democratic Republic of the Congo, RoC = Republic of Congo.		

**Table 2:** Completeness of epidemiological details among the 117 Ebola virus transmission occurrences.

	**Spread order (%)**	**Source (%)**	**Onset timing (%)**	**End timing (%)**	**Case data (%)**
Complete	51	69	32	6	27
Partial	44	2	41	0	21
Unknown	5	29	27	94	52
Spread order is considered complete if the order of each occurrence in their respective outbreaks is known, partial if their order could not be distinguished from every occurrence in each outbreak and unknown if their order was unknown in the outbreak. Source was considered complete if spread could be linked to one source occurrence, partial if spread is known to have come from one of a number of source occurrences and unknown if source was unidentifiable (index cases excluded). Both onset and end timing were considered complete if day, date and year was known, partial if just month and year was known. Case data was considered complete if the number of cases and deaths was known, partial if either cases or deaths was known.					
