# Supplementary material for: A comprehensive database of the geographic spread of past human Ebola outbreaks
Source: Sci Data. 2014 Oct 23;1:140042. doi: 10.1038/sdata.2014.42 (PMC4432636; doi:10.1038/sdata.2014.42)
Supplement: Supplementary Figures [file sdata201442-s1.pdf]

Supplementary figures 1-24: Ebola outbreak spread maps

Table of contents:

1. Figure 1: Sudan, 1976……………………………………………………………………………..………..1
2. Figure 2: DRC, 1976……………………………………………………………..………………………2
3. Figure 3: DRC, 1977……………………………………………………………………………..………..3
4. Figure 4: South Sudan, 1979……………………………………………………………..……………4
5. Figure 5: Côte d'Ivoire, 1994…………………………………..……………………..………………5
6. Figure 6: Gabon, 1994…………………………………………………………………..……………….6
7. Figure 7: DRC, 1995……………………………………………………………………………..……….7
8. Figure 8: Gabon, 1996…………………………………………………………………..……………….8
9. Figure 9: Gabon, 1996b……………………………………………………………………….…………9
10. Figure 10: Uganda, 2000……………………………………………………………………..………10
11. Figure 11: Gabon, 2001……………………………………………………………………………….11
12. Figure 12: Gabon, 2001b……………………………………………………………………..………12
13. Figure 13: RoC, 2003……………………………………………………………………………..……13
14. Figure 14: South Sudan, 2004………………………………………………………………………14
15. Figure 15: RoC, 2005……………………………………………………………………………..……15
16. Figure 16: DRC, 2007…………………………………………………………………………………..16
17. Figure 17: Uganda, 2007……………………………………………………………………………17
18. Figure 18: DRC, 2008…………………………………………………………………………………..18
19. Figure 19: Uganda, 2011……………………………………………………………………………..19
20. Figure 20: DRC, 2012…………………………………………………………………………………..20
21. Figure 21: Uganda, 2012……………………………………………………………………………..21
22. Figure 22: Uganda, 2012b……………………………………………………………………………22
23. Figure 23: All outbreaks……………………………………………………………………………..23

Figure legends. Square symbols indicate point locations and circular and irregular shaped objects represent polygons. Arrows indicate order of spread. Where spread order is known, numbers are indicative of the order of spread. Arrows sharing the same number indicate that it was not possible to distinguish which spread happened first. DRC = Democratic Republic of the Congo, RoC = Republic of Congo, CFR = case fatality rate.

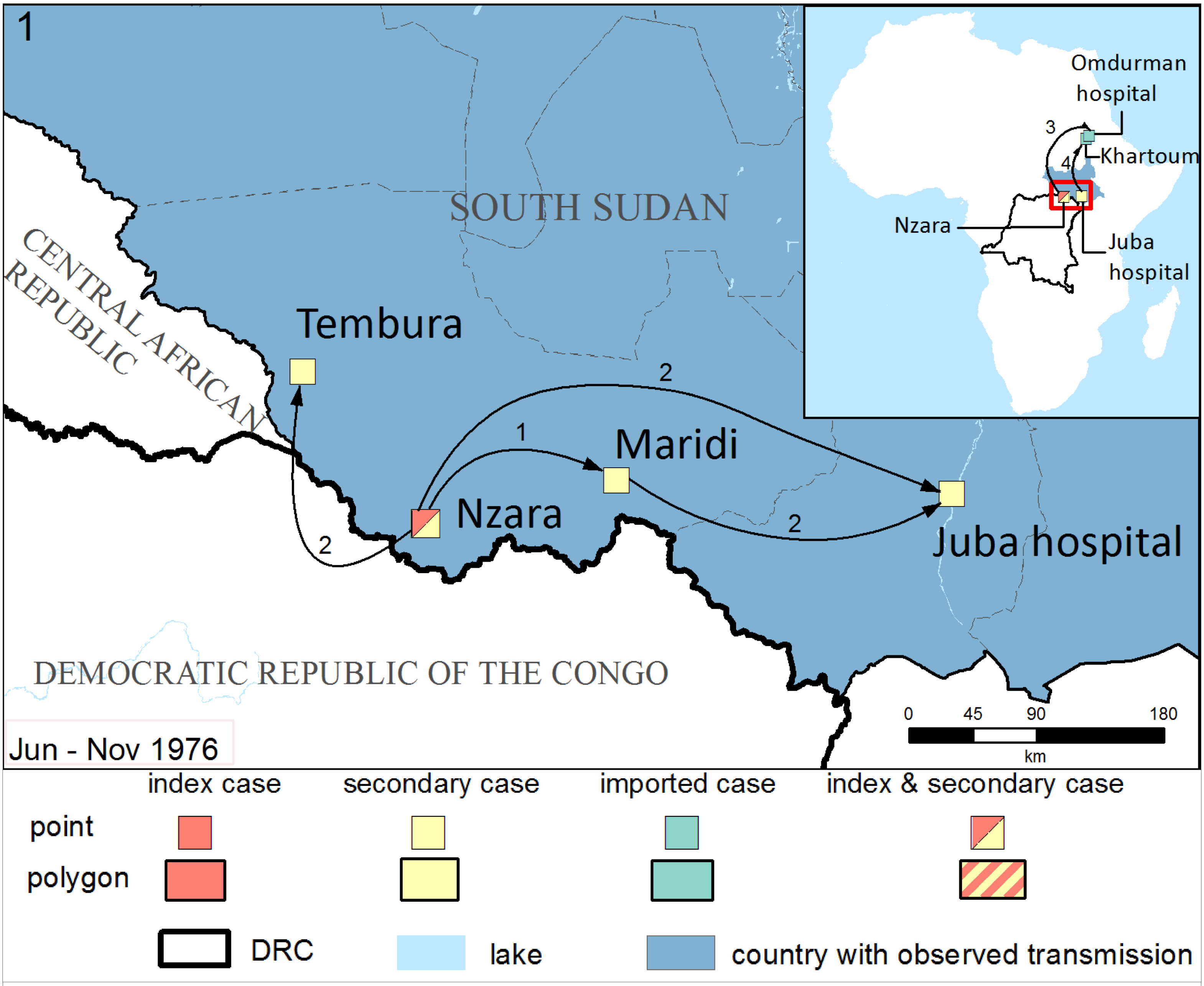

The first reported cases of Sudan Ebola virus were in three workers at a cotton factory in Nzara, in close proximity to three game reserves. The method of acquisition was unknown. The first secondary cases arose in Nzara infecting a total of 67 people who were primarily family members of the factory workers. Further secondary transmission clusters emerged in Maridi following spread from Nzara due to seeking treatment, after which further cases occurred in Juba due to patients who were referred. Additional cases from Maridi were also referred directly to Juba making the source of infection in Juba difficult to identify. Secondary transmission also emerged in Tembura due to a patient seeking family care, although the source of this infection is unknown. Imported cases from Juba to Khartoum and from Nzara to Omdurman were also reported following a patient seeking treatment and a referral for diagnosis respectively (see inset). The principal mode of transmission in this outbreak was initially familial, although in Maridi secondary transmission arose through nosocomial transmission. Seeking of treatment was the principal cause of geographic spread.

The first index case became ill on the 27 June 1976 before the first secondary cases in July and subsequent secondary transmission clusters from August to October. Cases peaked in September (138 cases, 65 deaths). The final case was reported on 25 November 1976. Imported cases in Omdurman and Khartoum were reported in August and September, respectively.

Overall, 284 cases were reported with 151 deaths giving a CFR of 53.2%. This figure varied in different locations: Nzara (67,31,46%), Maridi (213,116,55%), Tembura (3,3,100%), Juba (1,1,100%).

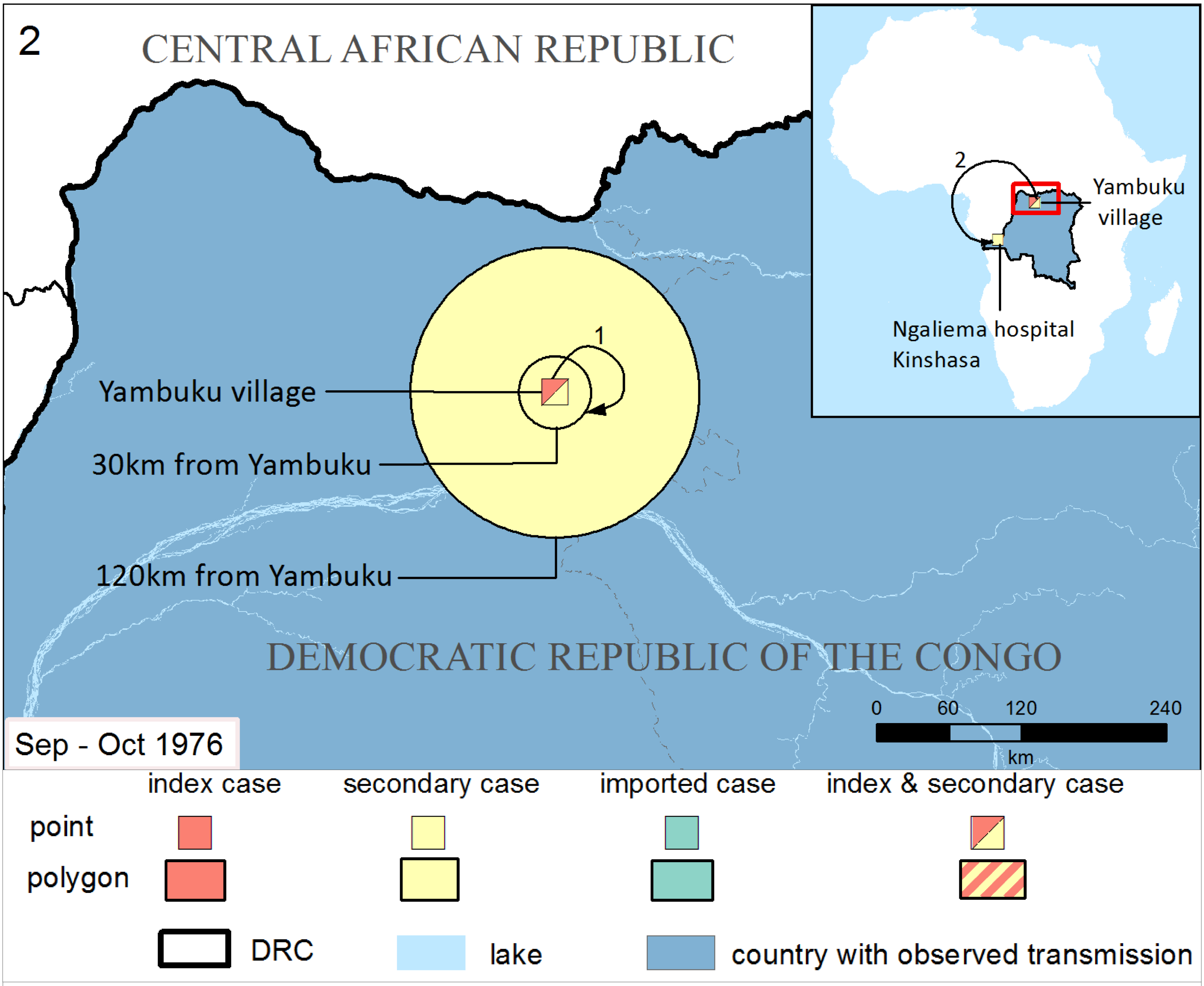

It is suspected that the index case of Zaire Ebola virus arose after a resident consumed bush meat purchased from a regional market at his home in Yambuku. The first secondary cases arose in the Yambuku Mission Hospital through nosocomial transmission. Following this, secondary transmission clusters occurred within a 30km radius due to radial spread through the community. A focal secondary transmission cluster also emerged at Ngaliema hospital, Kinshasa, following long-distance spread from Yambuku by a nurse seeking care (see inset). By the end of the outbreak radial spread of transmission infected 55 of the 250 villages within 120km of Yambuku, as defined by the most northerly (Abumombazi) and southerly (Bumba) infected villages. The principal mode of transmission in this outbreak was through nosocomial transmission at the focal site of Yambuku Mission Hospital, but the mode of the brief and more limited transmission in the surrounding villages was unknown. The index case was reported on 1 September 1976 before the first secondary cases in September and subsequent secondary transmission clusters from September to October. Cases peaked in September (201 cases).
The final case was reported on 24 October 1976. Overall 318 cases were reported with 280 deaths, giving a CFR of 88.1%.

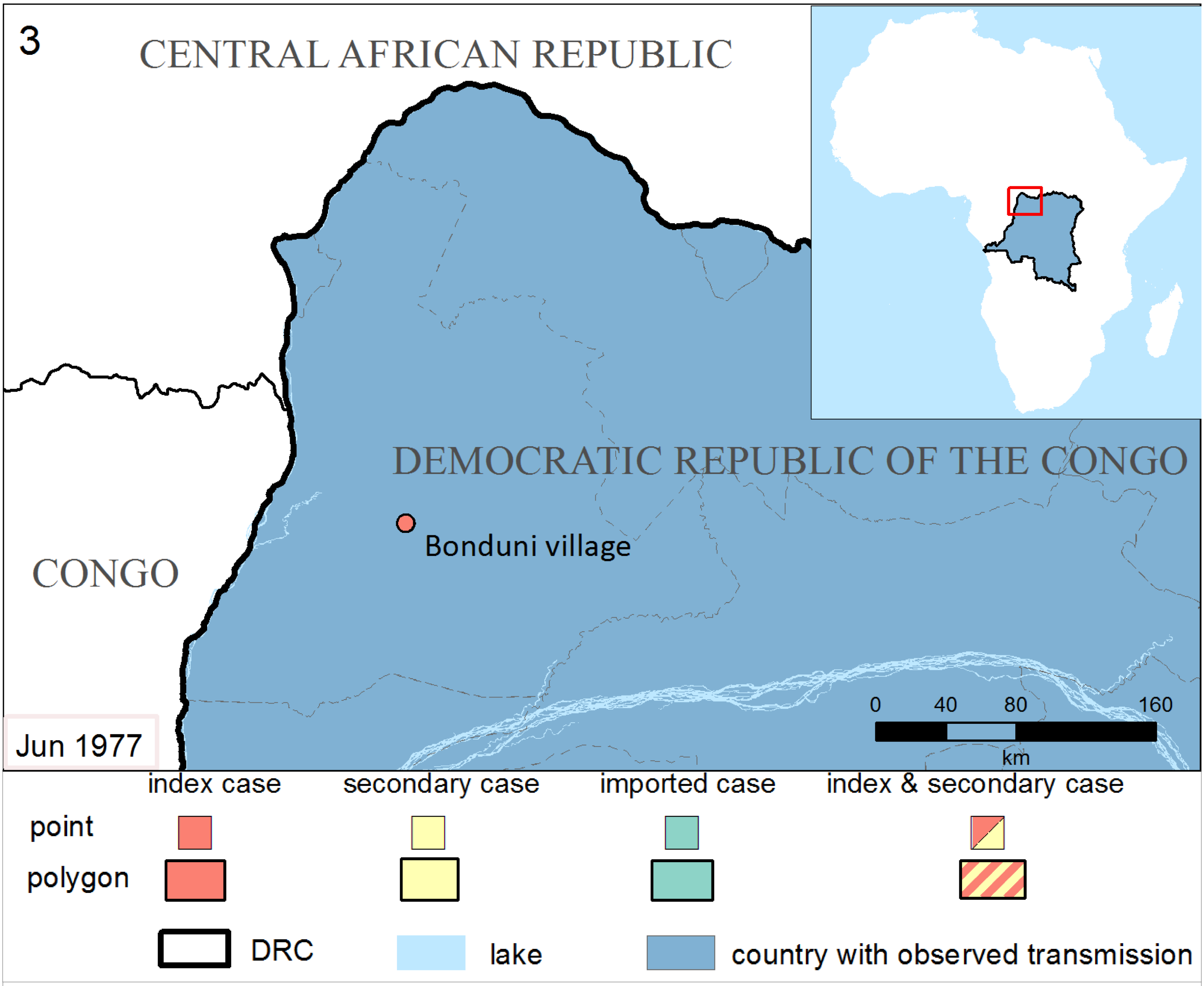

The first and only reported case of Zaire Ebola virus was in a nine-year-old girl from the village of Bonduni. The method of acquisition was unknown and no subsequent human cases were detected.
The index case was reported in June 1977 at the Tandala Mission Hospital and the patient died 28 hours after admission (CFR 100%).

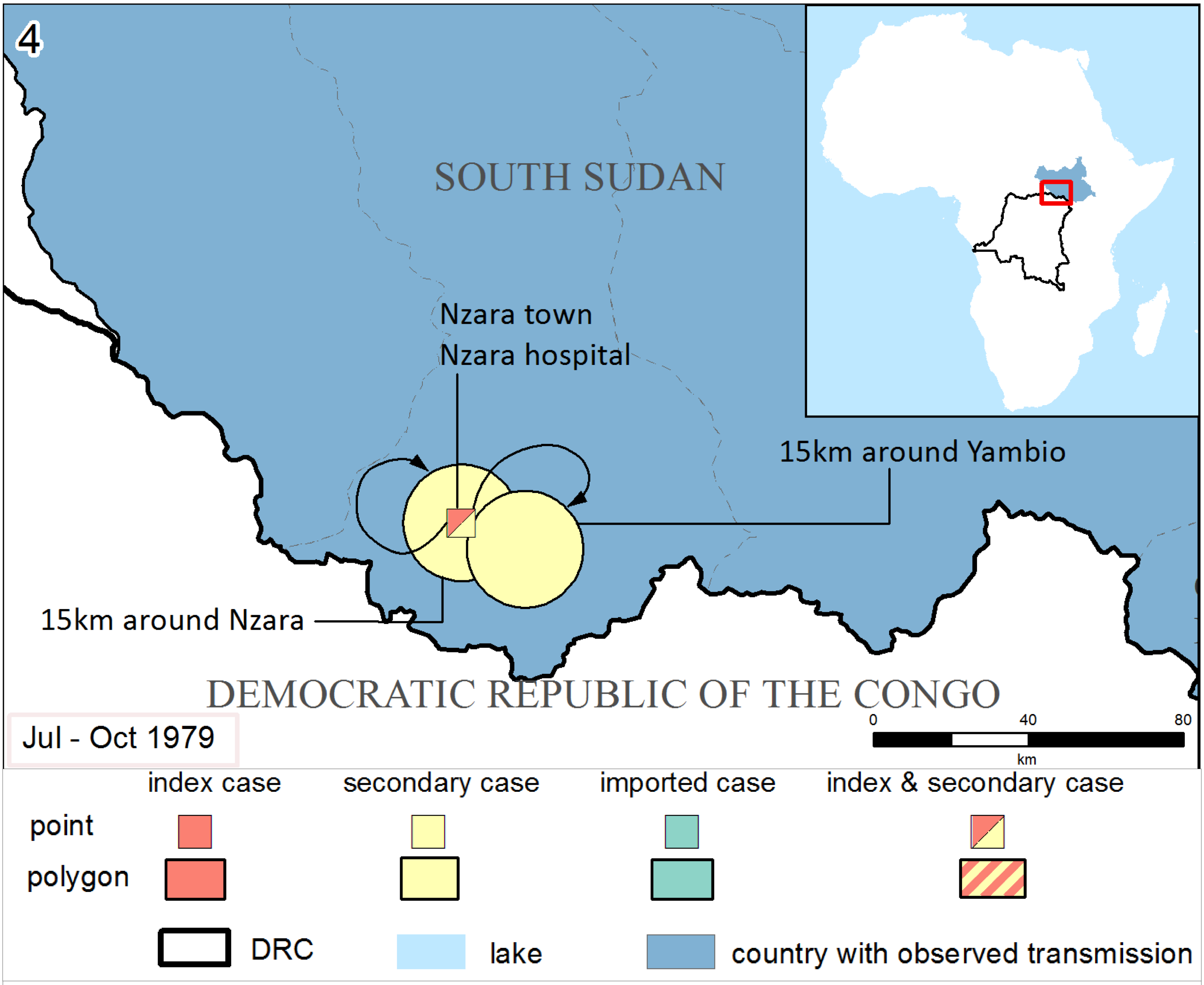

The first reported case of Sudan Ebola virus was in a textile factory worker in Nzara. The method of acquisition was unknown. The first secondary cases arose in Nzara hospital, infecting a total of 34 members of five separate families. Further secondary transmission clusters emerged within 15km of Nzara and within 15km of Yambio following close contact with the first wave of hospitalised cases. The principal mode of transmission in this outbreak was initially nosocomial, followed by familial transmission through home care for sick relatives.

The index case presented symptoms on 31 July 1979 before the first secondary cases in August and subsequent secondary transmission clusters from August to October. Cases peaked in September (17 cases, 8 deaths).

The final case was reported on 6 October 1979. Overall, 34 cases were reported with 22 deaths giving a CFR of 64.7%.

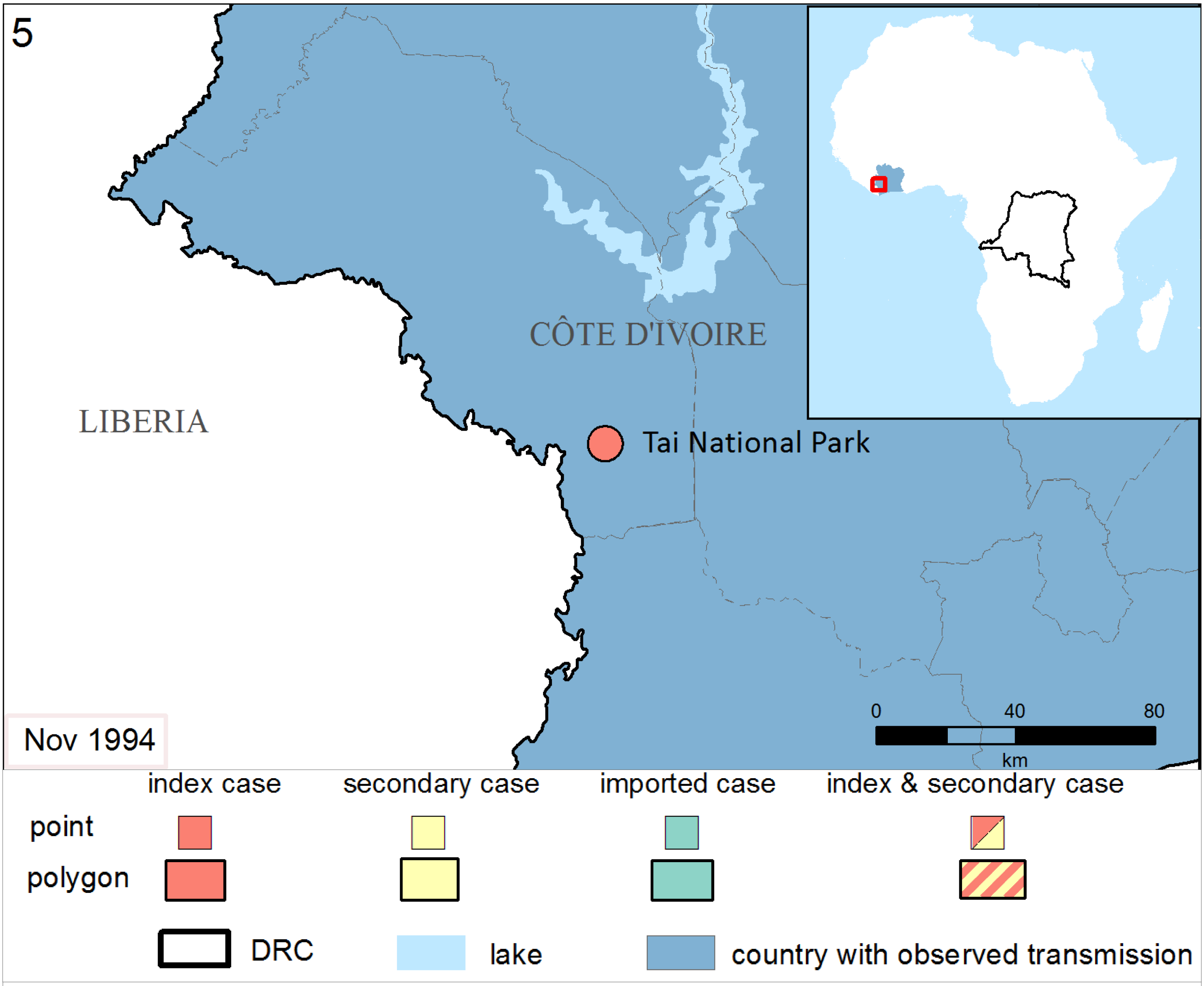

The index case of Tai Forest Ebola virus arose though the autopsy of an Ebola diseased chimpanzee. The index case presented symptoms on 24 November 1994 and was hospitalised on 26 November. The patient was later evacuated to Switzerland, where they recovered.

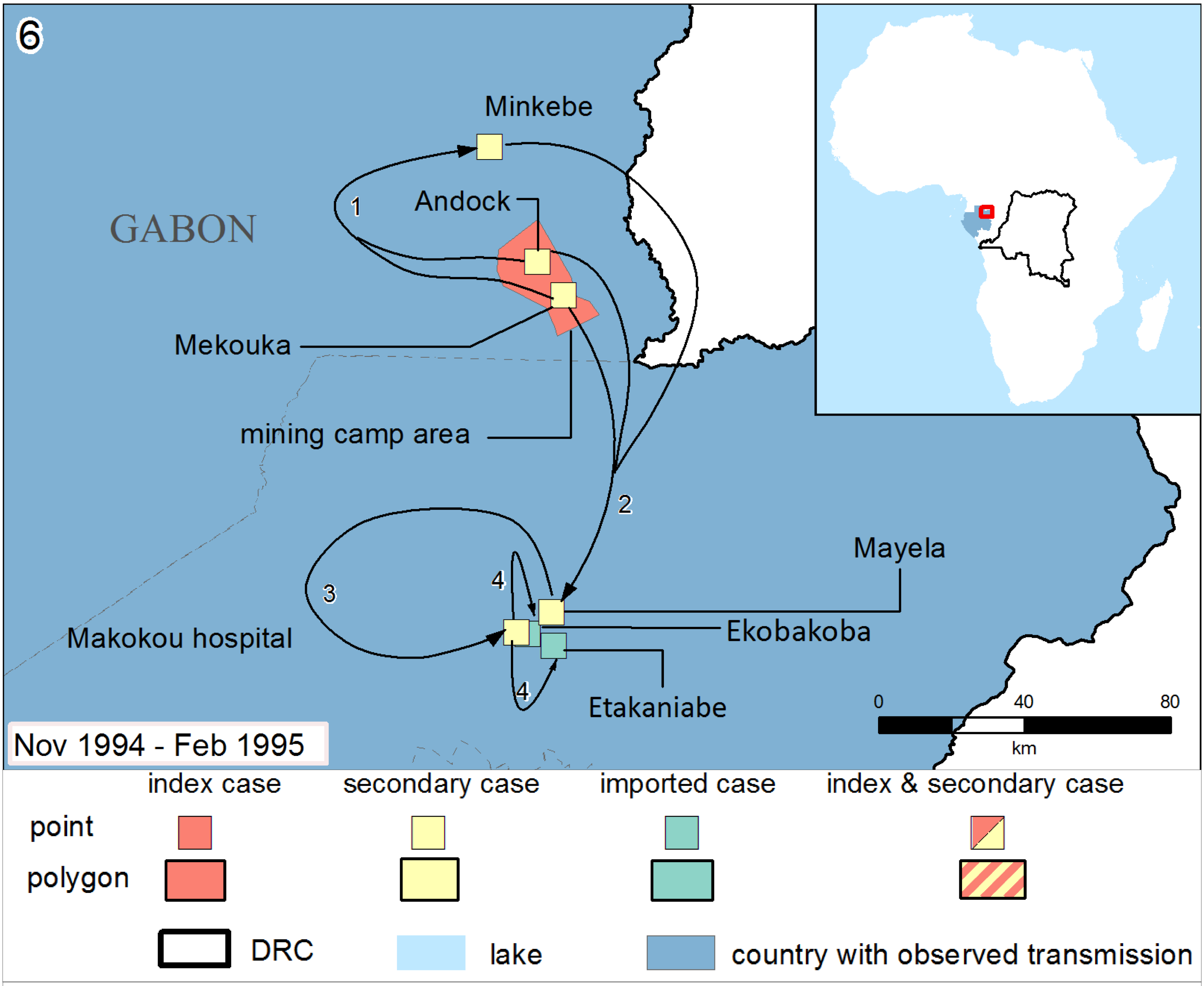

The first reported cases of Zaire Ebola virus were in miners from the Mekouka and Andock encampments, suspected to have contracted the infection in the surrounding area. The method of acquisition was unknown. The first secondary cases arose within these two encampments and then spread to the Minkebe camp. Further secondary transmission clusters emerged in Mayela then Makokou general hospital after 32 patients from the forest encampments sought treatment. Cases were also reported in Ekataniabe and Ekobakoba who had recent travel histories to Makokou general hospital. The principal modes of transmission were among workers at first, followed by nosocomial in Makokou general hospital and familial in Mayela (connected by a single traditional healer). The initial case was reported on 13 November 1994 before secondary transmission clusters occurred from the end of January to February 1995. Cases and deaths peaked in December (26 cases, 14 deaths (53.8% CFR)).
The final case was reported on 9 February 1995 in Ekobakoba. Overall, 49 cases were reported with 30 deaths, giving a CFR of 61.2%.

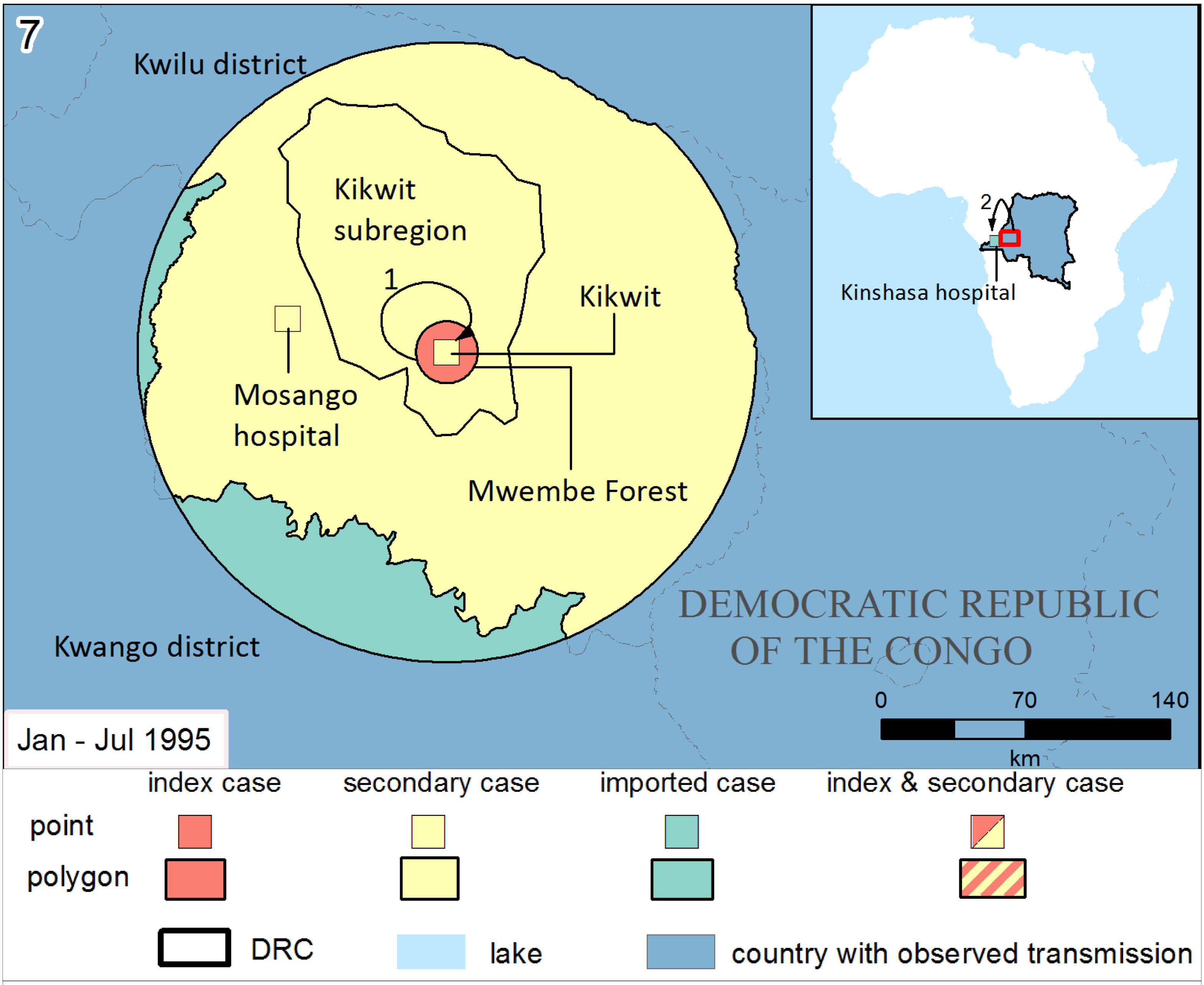

It is suspected that the index case of Zaire Ebola virus arose though exposure to the natural reservoir at a charcoal pit on a farm 15km from Kikwit. The first secondary cases were in immediate and extended family members, acquired via care for sick relatives and traditional burial practices in Kikwit and three surrounding villages. The outbreak then spread throughout Kikwit and Kikwit subregion and then as far as 120km from Kikwit, remaining confined to Kwilu district. Further secondary transmission clusters emerged in Mosango hospital following spread from patients seeking treatment or being referred from other healthcare centres. From the hospital the outbreak then spread into the surrounding community and imported cases were reported in a hospital in Kinshasa and Kwango district (see inset) after infected individuals sought treatment. Familial care for sick relatives and burial rituals for these initial cases led to further secondary transmission, whereby the disease then propagated throughout the community.

The index case was reported on 6 January 1995 before subsequent secondary transmission clusters from March to July. Cases peaked in May (173 cases). The final case was reported in July, eventually dying on 16 July.

Overall, 315 cases were reported with 250 deaths giving a CFR of 79.4%.

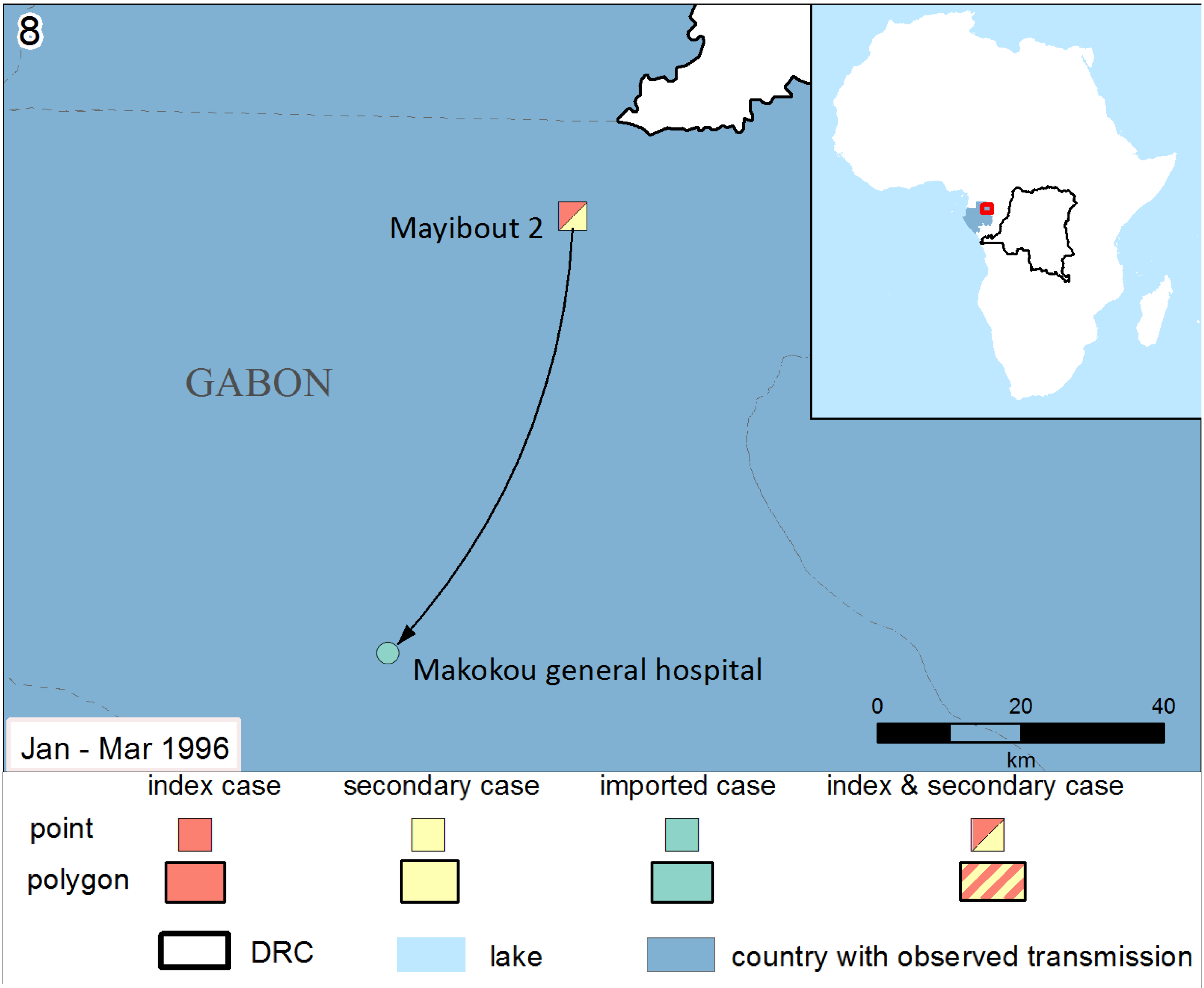

It is suspected that the index case of Zaire Ebola virus arose when eighteen people skinned and chopped a chimpanzee cadaver in the village of Mayibout 2. The first secondary cases arose in Mayibout 2 through contact with the index case during their traditional burial ceremonies. Imported cases in Makokou General Hospital were also reported after all eighteen index and first wave secondary cases sought treatment. The principal mode of transmission in this outbreak was familial.

The index case(s) were reported on 31 January 1996 before the first secondary cases in February. The final case was reported on 3 March. Imported cases in Makokou General Hospital were reported between the 12 and 18 February.

Overall, 31 cases were reported with 21 deaths, giving a CFR of 67.7%.

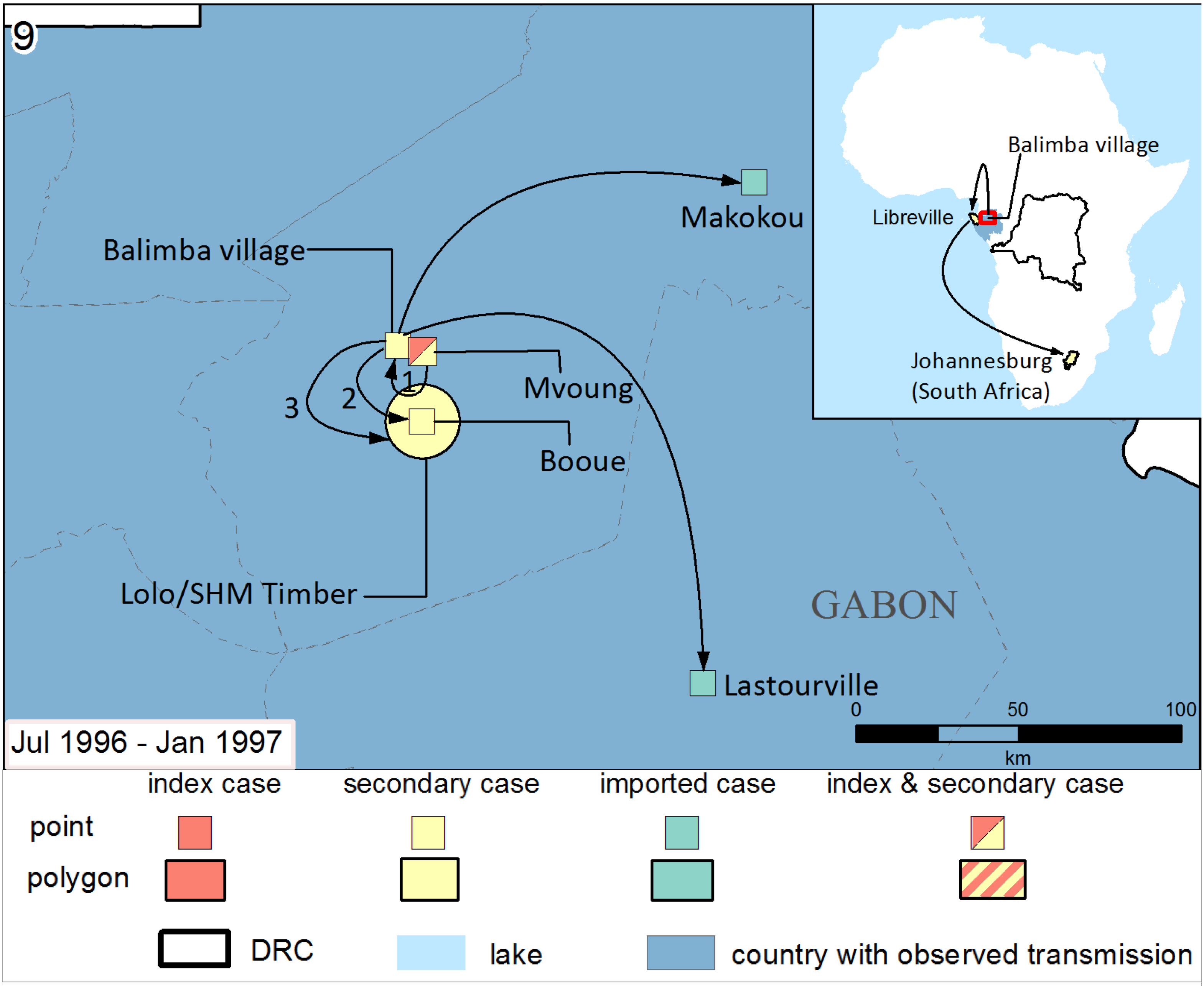

The index case of Zaire Ebola virus likely came from one of three infected hunters in a logging camp near Mvoung. The timing of infection makes it difficult to distinguish index cases from secondary cases during the early stages of this outbreak, but it is likely that the first secondary cases emerged amongst the hunters who then sought treatment from a traditional healer in Balimba. After falling ill, the traditional healer from Balimba sought treatment in Booue, where the disease then radially spread through the communities in the surrounding areas. A further secondary transmission cluster emerged in Libreville (see inset) after patients from Balimba sought treatment there. In Libreville one doctor became infected and flew to Johannesburg, South Africa for treatment before receiving a diagnosis of Ebola. Limited further nosocomial transmission (1 case) occurred upon his arrival in Johannesburg. Imported cases in Makokou General Hospital and Lastourville were also reported after patients from Balimba sought treatment. No clear principal mode of transmission was observed for the early stages of the outbreak, but in Libreville secondary transmission mainly arose through nosocomial transmission.

The index case was reported on the 13 July 1976 before the first secondary cases in September and subsequent secondary transmission clusters from September to January. Cases peaked in September and deaths peaked in October.

The final case was reported on 18 January 1997. Overall 60 cases were reported with 45 deaths, giving a CFR of 75%.

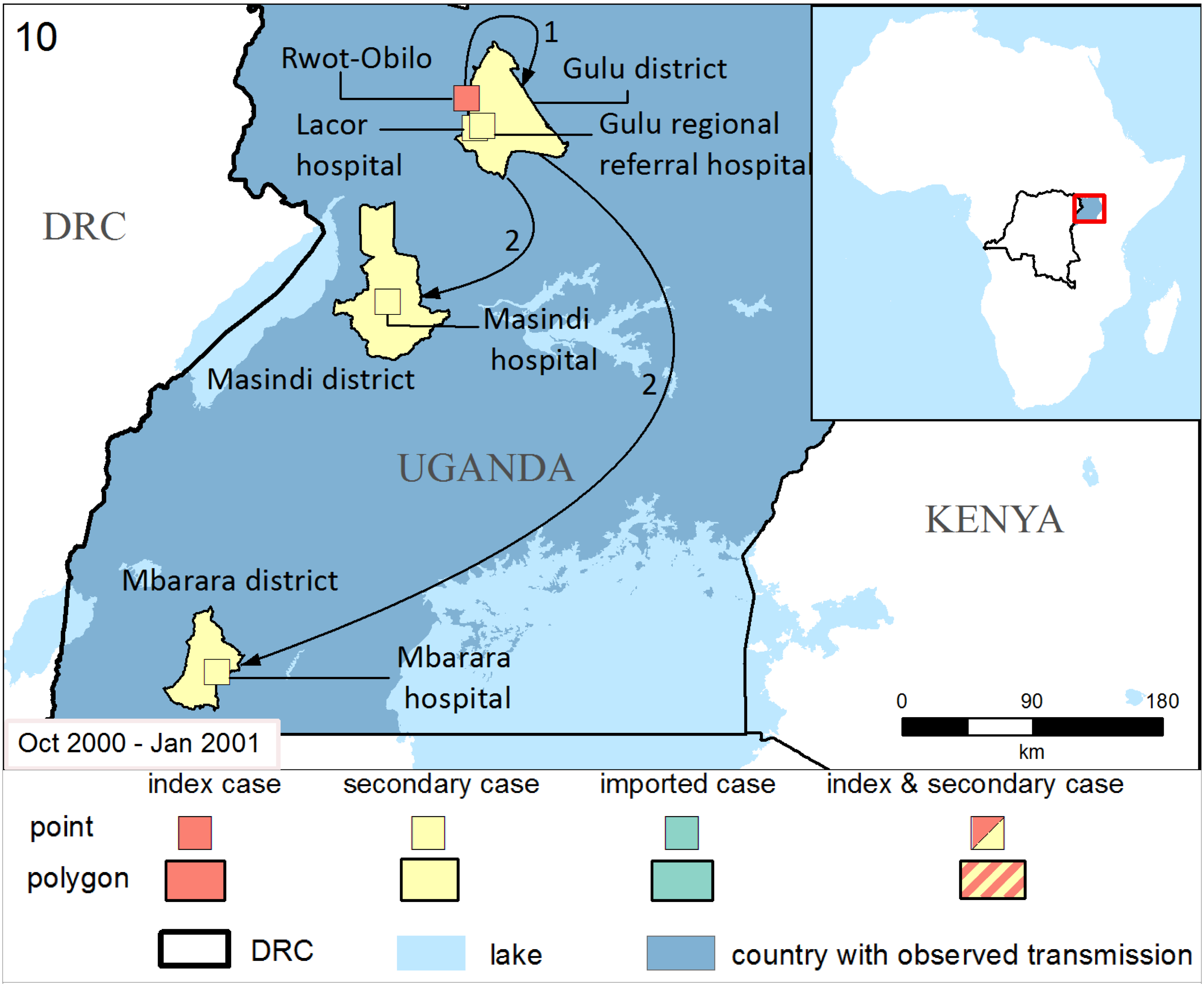

The first reported case of Zaire Ebola virus was suspected to have occurred in the remote village of Rwot Obilo in the far north of Gulu. The method of acquisition was unknown. The first secondary cases arose within Gulu district among people who attended a traditional burial ceremony involving washing of the corpse before burial. Further secondary transmission clusters emerged in Masindi and Mbarara districts after infected individuals from Gulu district arrived for unknown reasons. The principal modes of transmission were initially traditional burial practices followed by nosocomial transmission in the hospitals of Masindi and Mbarara.

The index case was reported to the Ministry of Health on the 8 October 2000, although it was suspected to have occurred on 30 August before the first secondary cases in September and subsequent secondary transmission clusters from September to January. Cases peaked in October. The final case was reported on 9 January 2001.

Overall, 425 cases were reported with 224 deaths, giving a CFR of 52.7%. This figure varied in different locations: Gulu District (393,203,51.7%), Masindi District (27,17,63.0%), and Mbarara Distrcit (5,4,80.0%).

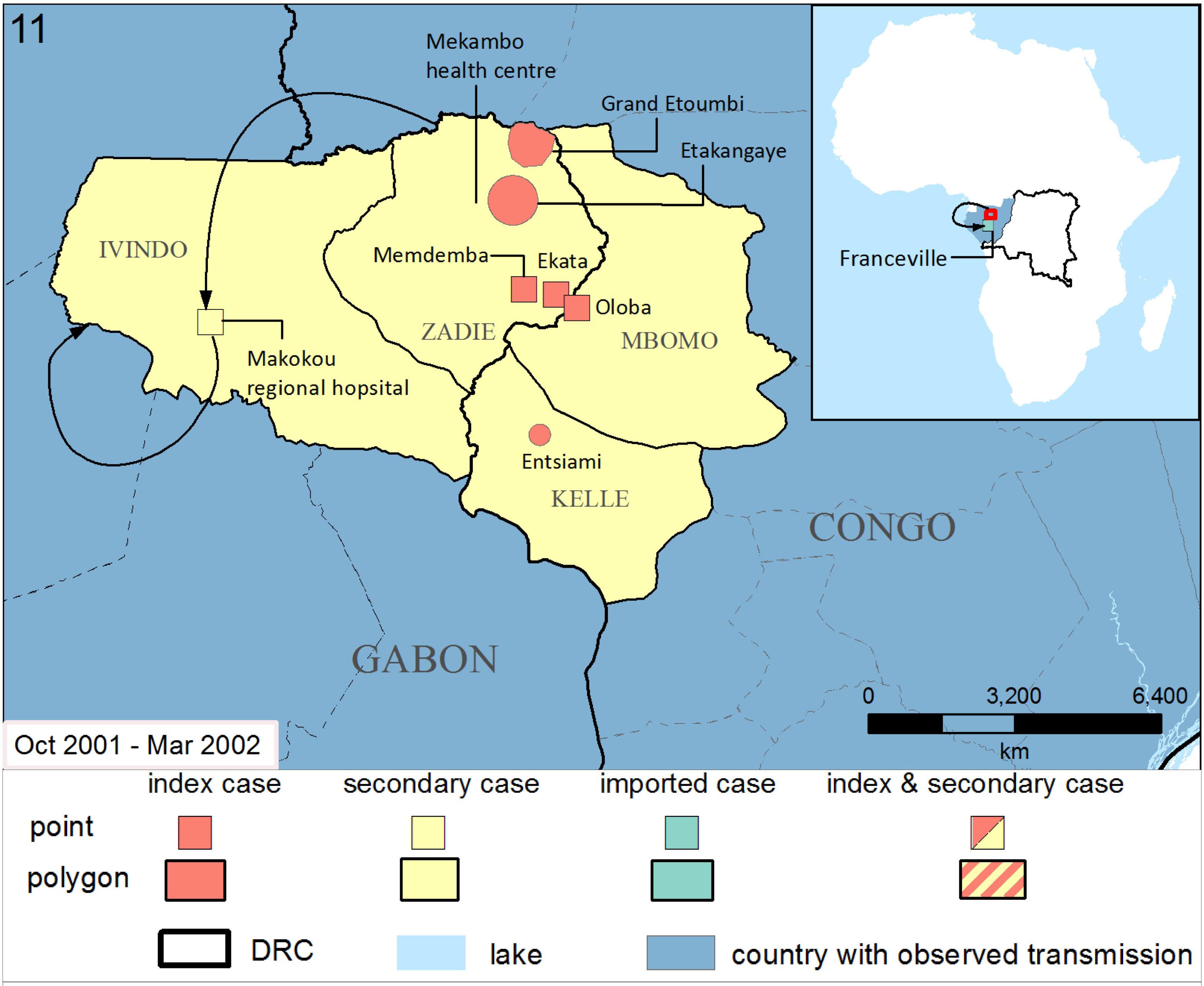

There is evidence for at least six different introductions of Zaire Ebola virus into human communities in an area straddling the RoC/Gabon border, each related to a hunting episode. The first secondary cases arose in La Zadie district in Gabon and Mbombo and Kelle districts in RoC through familial activities, including home nursing and traditional burial practices. Further secondary transmission clusters emerged in Ivindo district, Gabon following spread from La Zadie, when two patients were admitted to Makokou regional hospital. Imported cases occurred in Franceville (see inset) but no subsequent cases were reported. The principal mode of transmission in this outbreak was community based.

The first index case was reported on 21 October 2001 and the last on 23 February 2002 before the first secondary cases in November and subsequent secondary transmission clusters up until March 2002. Cases peaked in January.

The final case was reported on 18 March. In Gabon, in total, 65 cases were reported with 53 deaths, giving a CFR of 81.5%. Cases varied in different locations: La Zaide district (47) and Ivindo district (17). In RoC, there were a total of 59 cases reported with 44 deaths, giving a CFR of 74.6%. Cases varied in different locations: Mbomo district (33) and Kelle district (26).

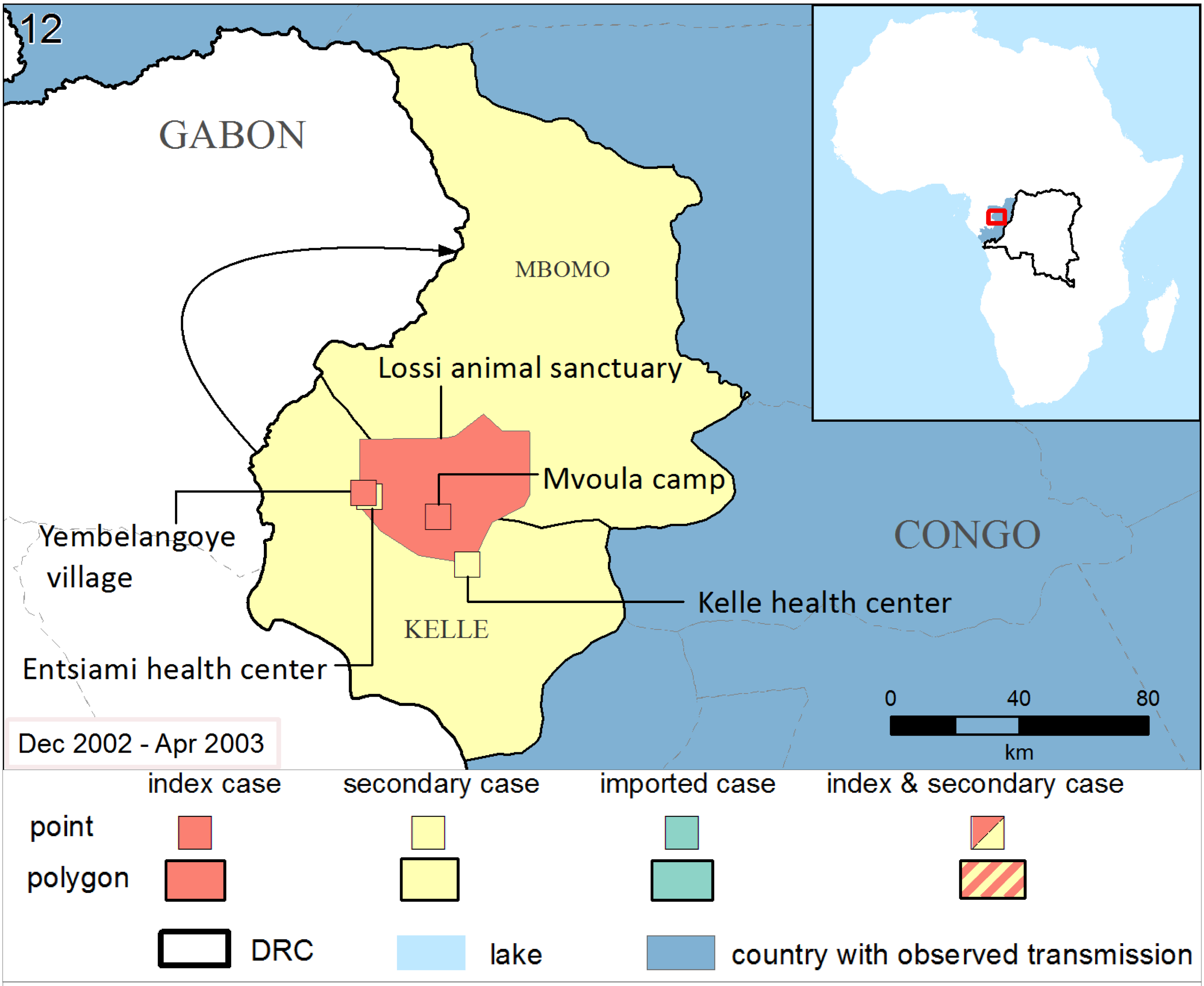

There is evidence for at least three different introductions of Zaire Ebola virus into human communities, each related to a hunting episode with exposure to gorillas and forest antelope carcases in the Lossi animal sanctuary. The first secondary cases then arose in limited clusters in two nearby healthcare centres followed by a wider outbreak in Kelle district through intra-family transmission, although the order of spread between these locations is unknown. Further secondary transmission clusters emerged in Mbomo district following spread from Kelle district. The principal mode of transmission in this outbreak was via community and familial contact. Nosocomial transmission did not play an important role in amplification of the epidemic.

The first index case was reported on the 21 December 2002 and the last on the 1 January 2003, before the first secondary cases in January and subsequent secondary transmission clusters until April 2003. Cases peaked in February. The final case was reported at the beginning of April.

Overall, 143 cases were reported with 128 deaths giving a CFR of 89.5%.

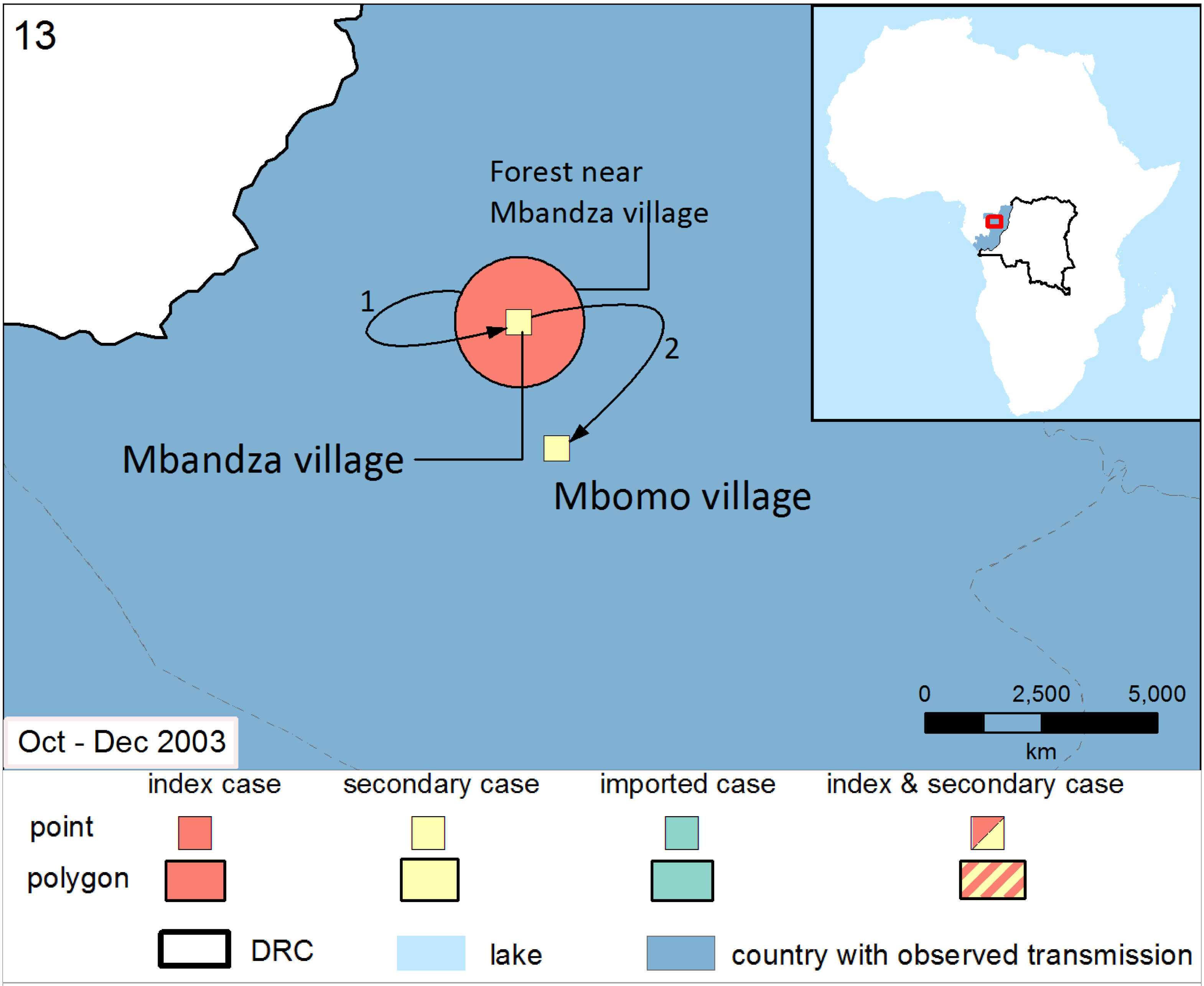

It is suspected that the index case of Zaire Ebola virus arose though exposure to the body fluids of a greater white-nosed monkey (*Cercopithecus nictitans*) that was killed during a hunting trip in a forest near Mbandza village. The first secondary cases arose in Mbandza village through familial transmission, which spread then causing further secondary transmission clusters in Mbomo village. The principal mode of transmission in this outbreak was familial, related to home nursing care and traditional burial practices.

The index case was reported on 11 October 2003 before the first secondary cases in October and subsequent secondary transmission clusters until December. Cases peaked in November (26 cases and 20 deaths). The final case was reported on 2 December 2003.

Overall, 35 cases were reported with 29 deaths giving a CFR of 82.9%.

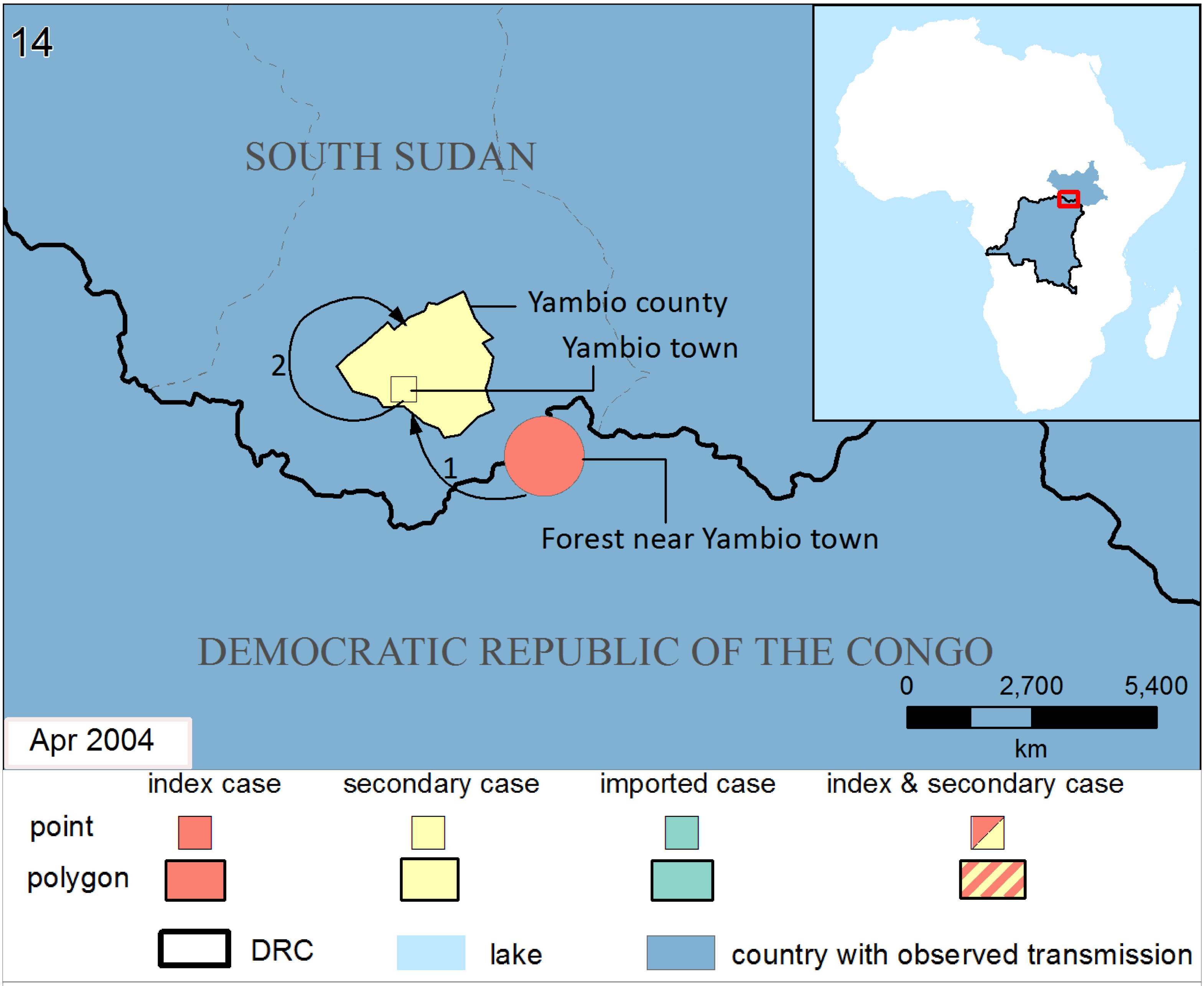

It is suspected that the index case of Sudan Ebola virus arose though exposure to baboon meat while hunting in a forest 40km south of Yambio in the DRC. The first secondary cases arose in Yambio town through community based transmission. Further secondary transmission clusters emerged in Yambio county following spread from the town. The principal mode of transmission in this outbreak was through familial and community contact.

The index case was reported on 15 April 2004 with cases peaking in April (13 cases). The final case died on 26 June.

Overall, 17 cases were reported with 7 deaths giving a CFR of 82.9%.

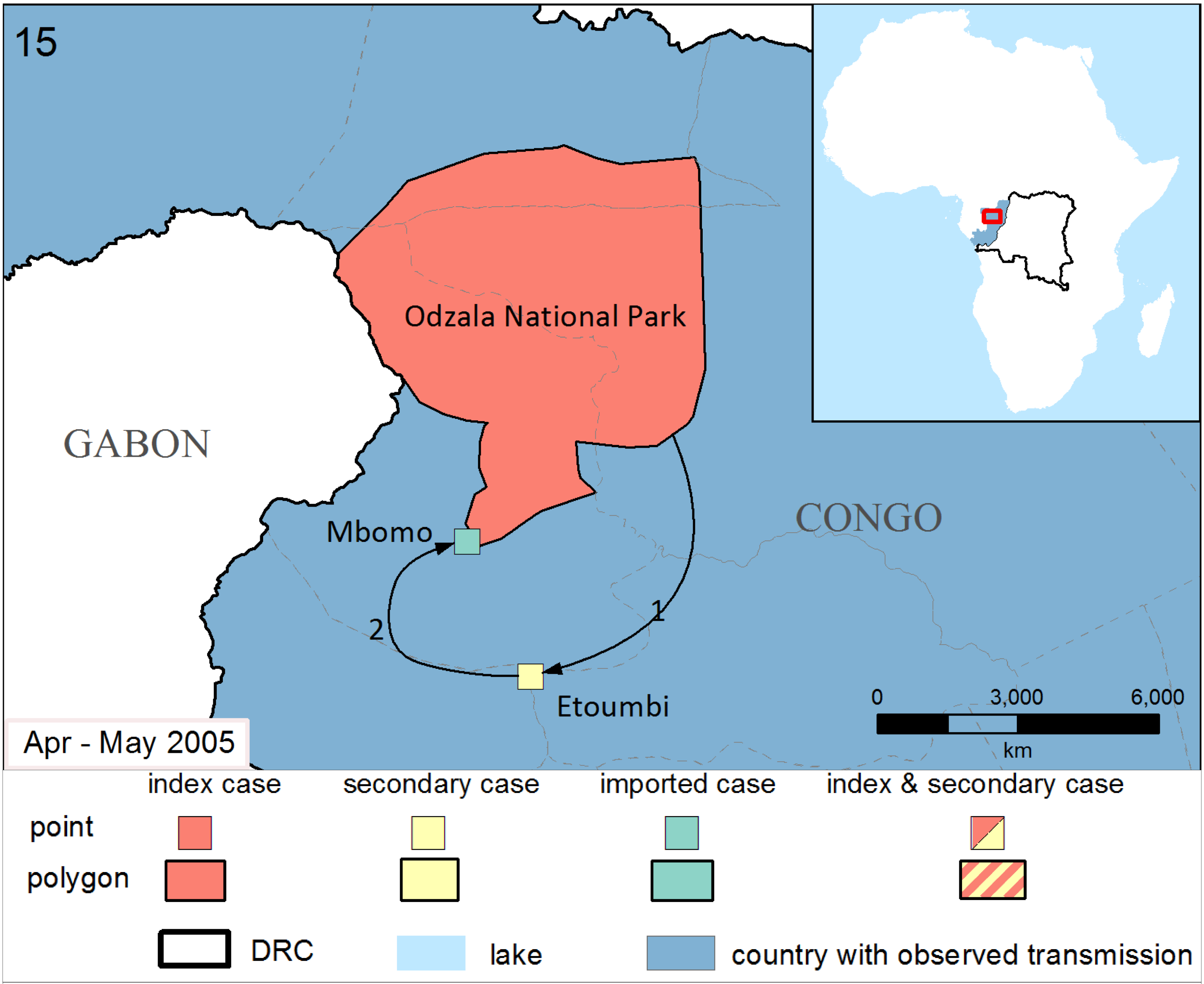

It is suspected that both index cases of Zaire Ebola virus arose during a hunting trip in Parc d'Odzala national park. The first secondary cases arose in Etoumbi through non-familial community contact and traditional familial burial practices. An imported case in Mbomo also resulted from attendance of the funeral ceremony of the index case in Etoumbi. The principal mode of transmission in this outbreak was through community contact and burial practices.

The index case was reported on 18 April 2005 and subsequent secondary transmission clusters occurred until May, when cases peaked. The final case was reported on 18 May 2005.

Overall, 12 cases were reported with 10 deaths giving a CFR of 83.3%.

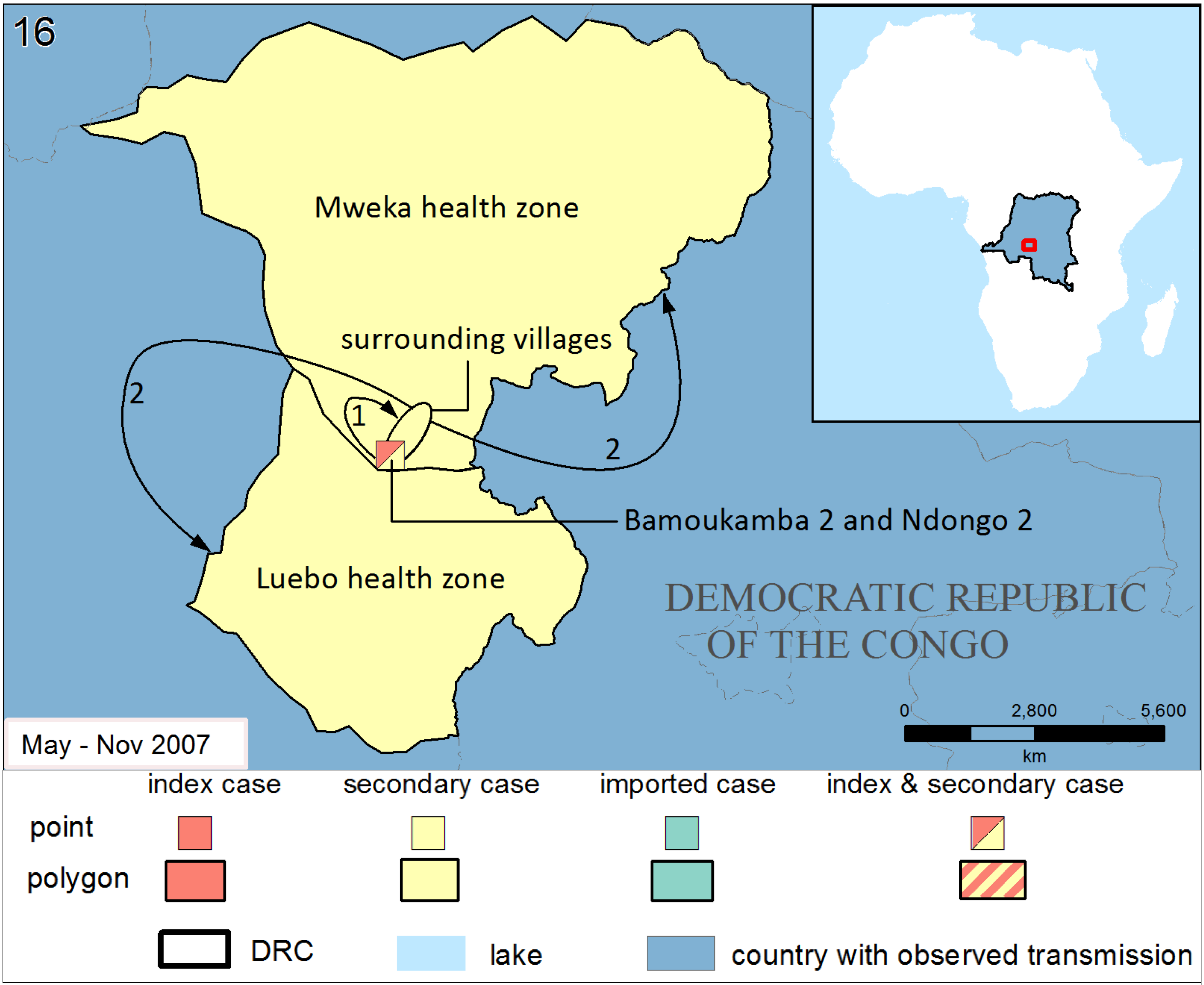

It is suspected that the index case of Zaire Ebola virus arose though direct contact with blood from a bat in Bamoukamba 2 village after purchasing the bush meat from a local market. The first secondary cases then arose in Bamoukamba 2 and Ndongo 2 villages through familial transmission. Further secondary transmission clusters emerged in the agglomeration of 10 villages situated on the north-south road linking Luebo and Mweka following spread from the case in Ndongo 2 village. The outbreak then spread throughout the Luebo and Mweka health zones. The principal mode of transmission in this outbreak was community driven.

The index case was reported to have been infected towards the end of May.

The final case was reported in November 2007. Overall, 264 cases were reported with 186 deaths, giving a CFR of 70.5%.

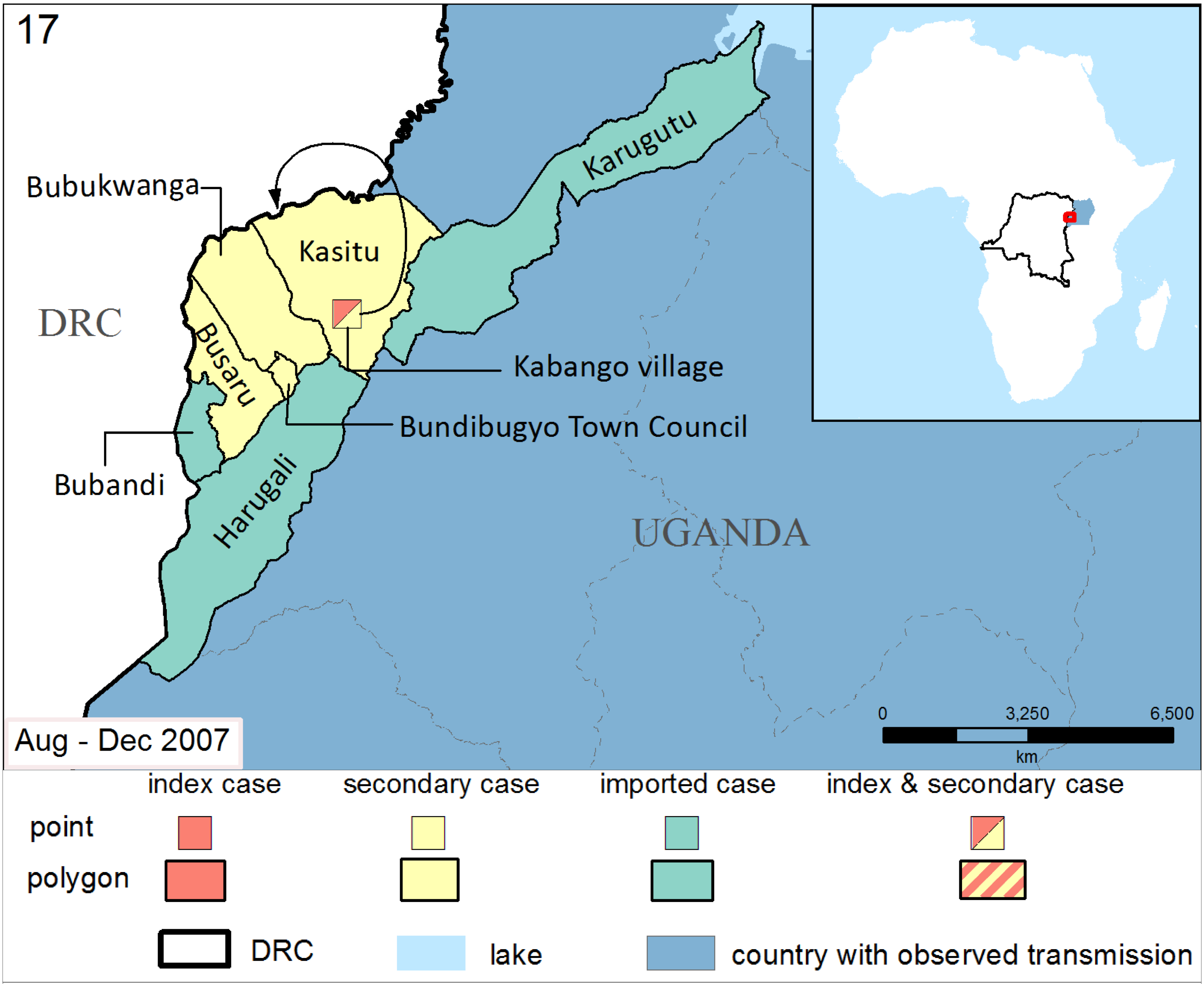

It is suspected that the index case of Bundibugyo Ebola virus was in a woman from Kabango village. Although the index case owned hunting spears, she denied any hunting activity and the method of acquisition is thus unknown. The first secondary cases arose in Kabango village, primarily in family members. Further secondary transmission clusters emerged in Kasitu subcounty following spread from Kabango. Subsequent secondary transmission clusters emerged in Bundibungyo town council and the Bubukwanga and Busaru districts, although with unknown origin. Imported cases in Harugali, Karugutu and Bubandi districts were also reported. The principal mode of transmission in this outbreak was community based.

The index case was reported on the 1 August 2007 before three subsequent secondary transmission clusters lasting around six weeks each. Cases peaked on 25 November and the final case was reported at the end of December 2007.

Overall, 116 cases were reported with 39 deaths giving a CFR of 33.6%. This figure varied in different locations: Kasitu subcounty (63/18/29%), Bundibungyo town council subcounty (25,8,32%), Bubukwanga subcounty (17,7,41%), Busaru subcounty (8,3,38%).

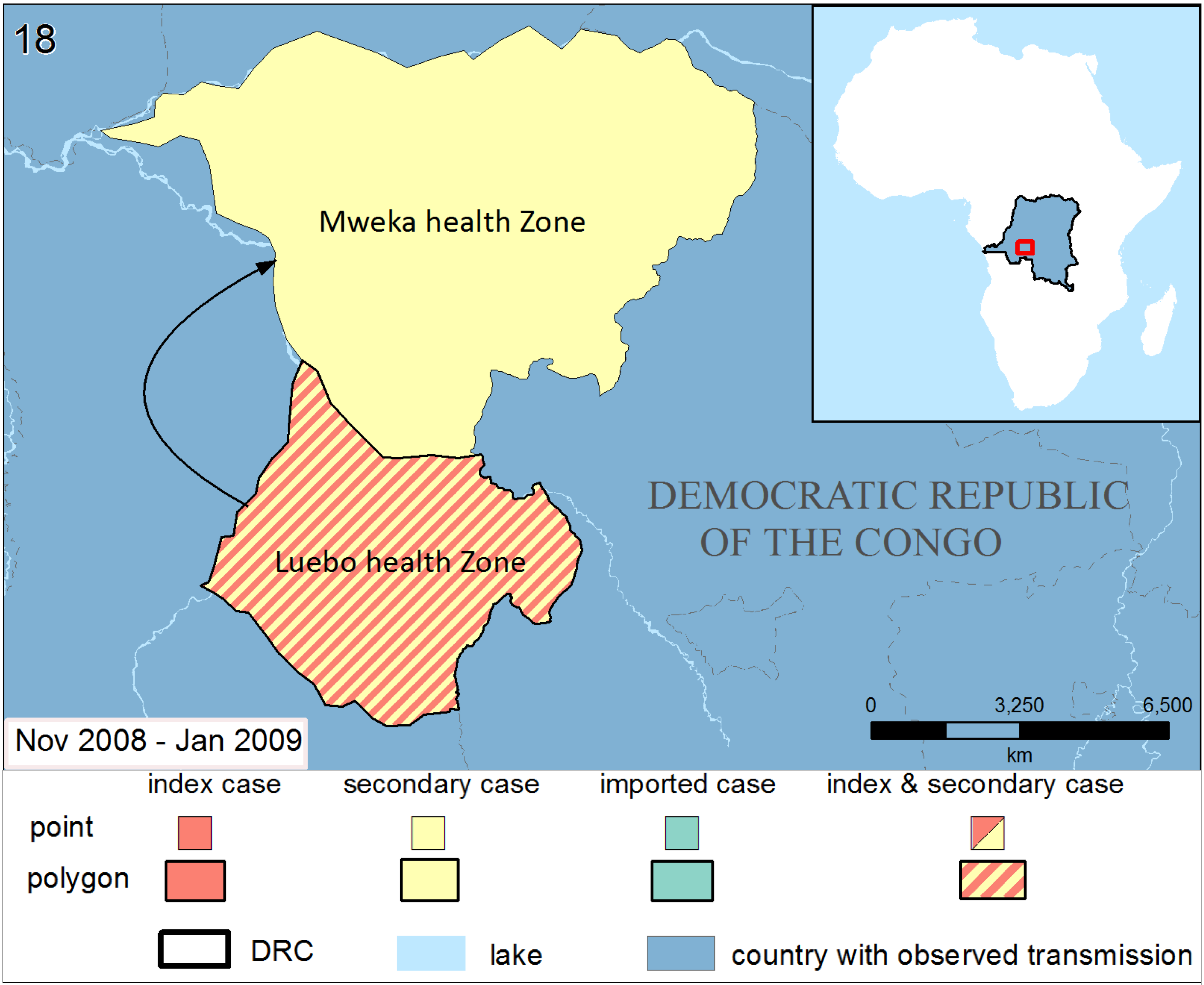

The first reported case of Zaire Ebola virus was in an 18-year old woman in the Luebo health zone. The method of acquisition was unknown. The first secondary cases then arose in 13 contacts in Luebo, then spreading to the Mweka health zone. No epidemiological investigation was conducted so the principal mode of transmission is unknown.

The index case was reported on 27 November 2008 with the last infected person dying on 1 January 2009.

Overall, 32 cases were reported with 15 deaths, giving a CFR of 46.9%.

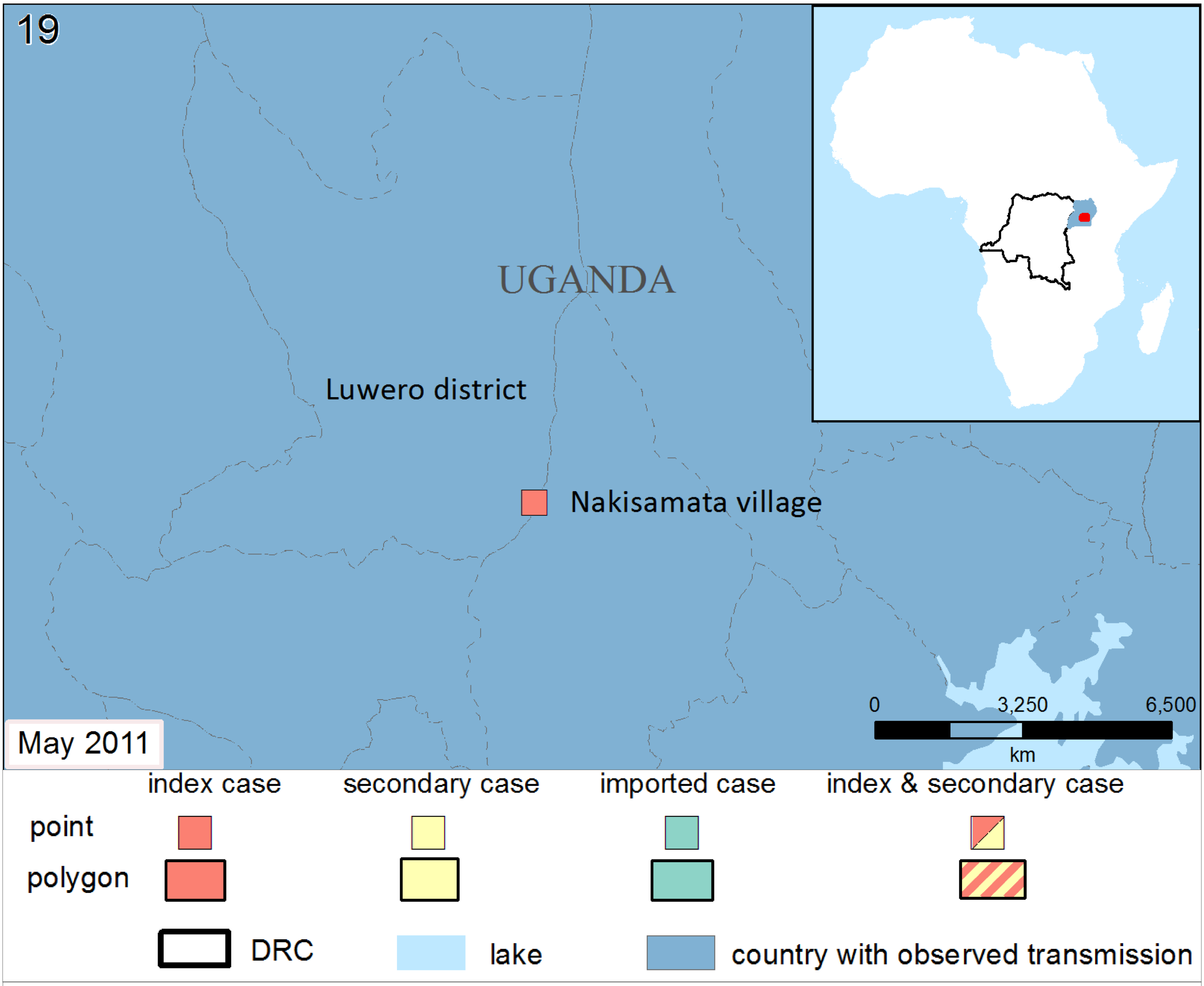

The only reported case of Sudan Ebola virus was in a 12-year old girl from Nakisamata village, Luwero district. The method of acquisition was unknown. She was admitted to Bombo hospital on 6 May 2011 and died three hours after admission (CFR 100%).

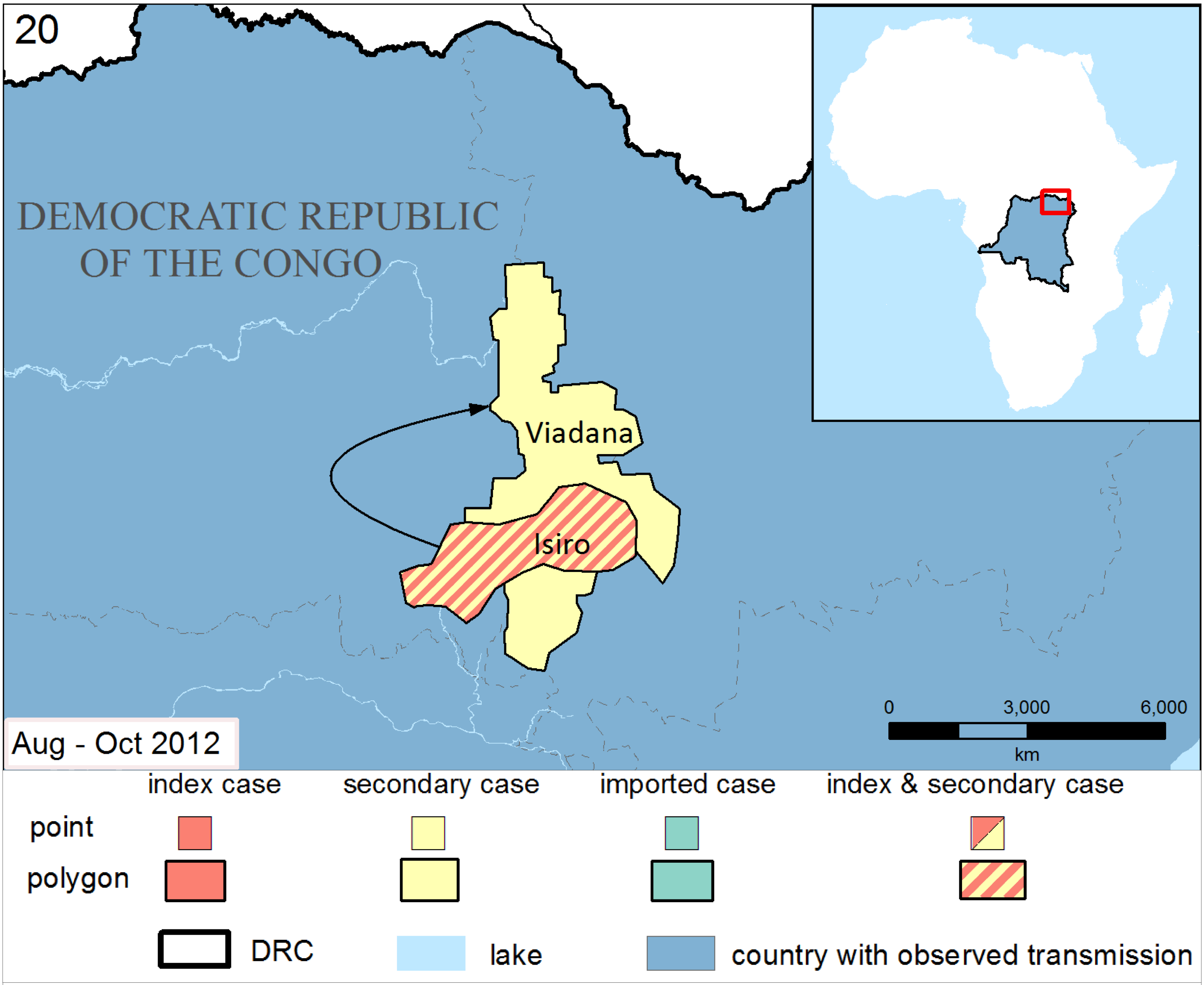

The first reported case of Bundibugyo Ebola virus was in the Isiro health zone. The method of acquisition was unknown. Secondary cases then arose in both the Isiro and Viadana health zones with cases among both the community and healthcare professionals.

The date of first laboratory confirmation was on 16 August 2012 and the last confirmed case was on 11 October 2012.

Overall, 36 confirmed cases were reported with 13 deaths, giving a CFR of 36.1%.

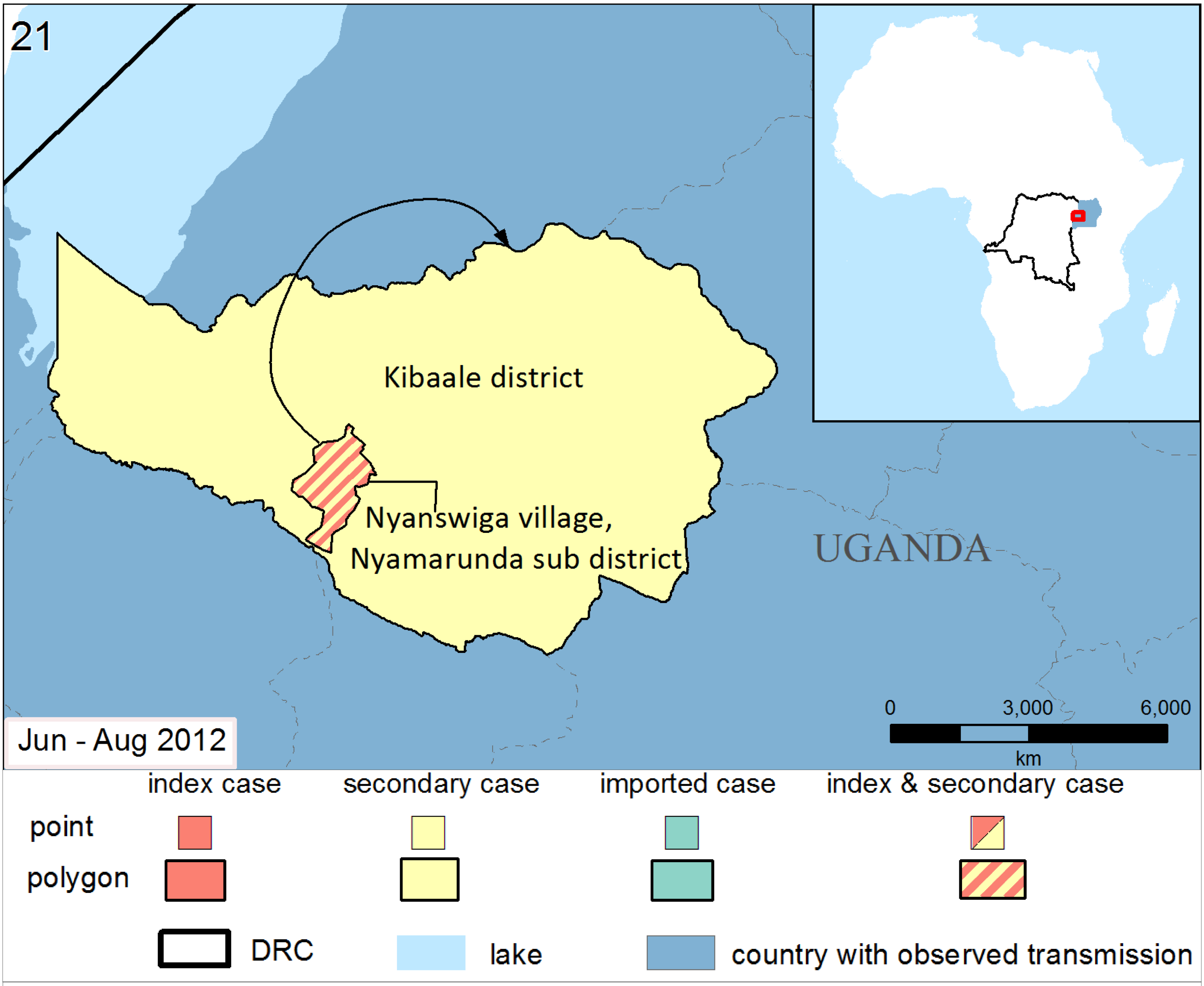

It is suspected that the index case of Sudan Ebola virus was in family from Nyanswiga village, Nyamarunda sub district. The method of acquisition was unknown. The first secondary cases arose in Nyanswiga village primarily among family members. Further secondary transmission emerged in the Kibaale district following spread from Nyanswiga village. The principal mode of transmission from this outbreak is unknown.

The index case was reported on 11 June 2012 and the final case was reported on 15 August 2012.

Overall, 11 confirmed cases were reported with 4 deaths, giving a CFR of 36.4%.

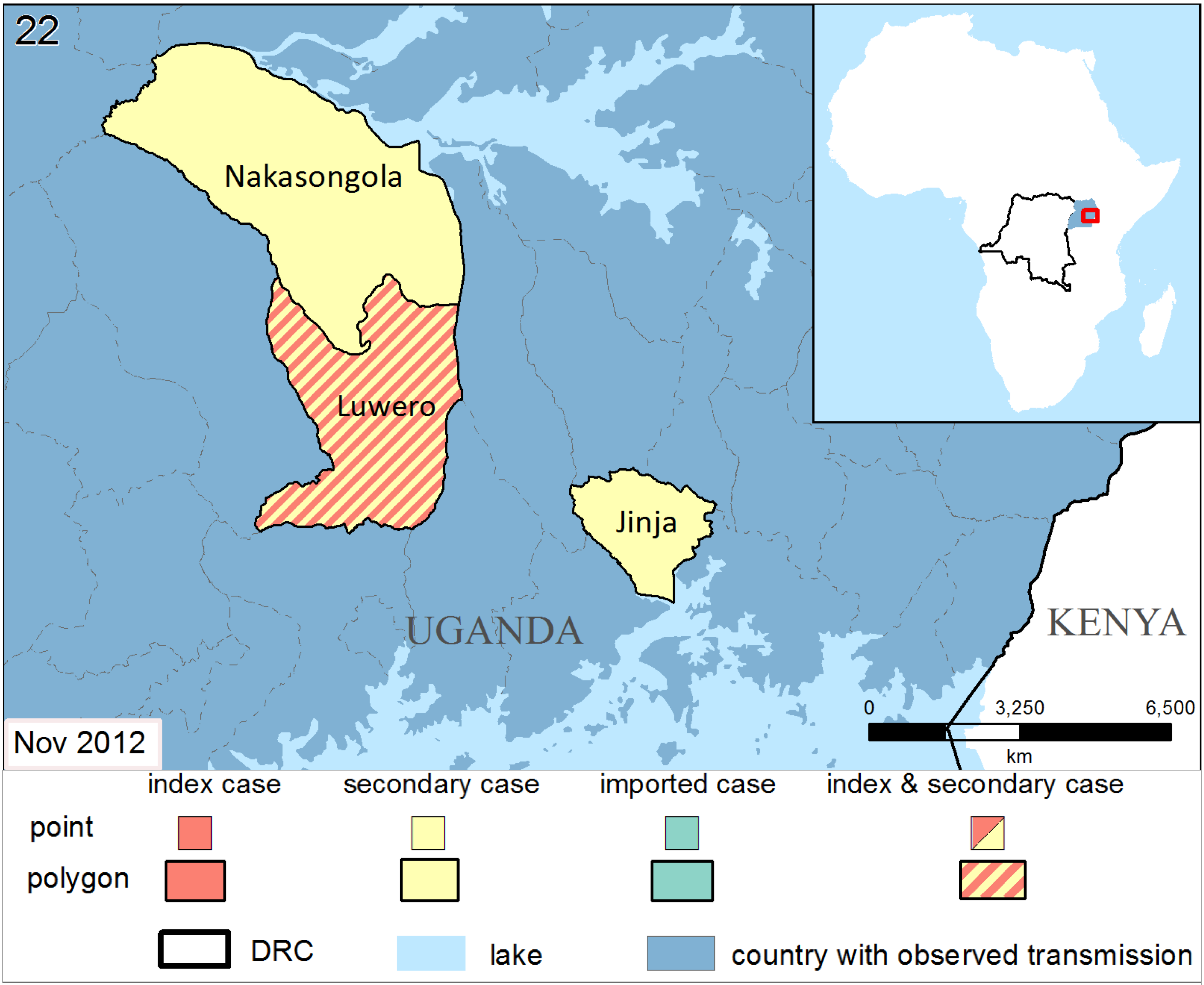

The first reported case of Sudan Ebola virus was in the Luwero district. The method of acquisition was unknown. Further secondary transmission emerged in Luwero and the nearby districts of Jinja and Nakasongola.

The limited outbreak began in November 2012 and was officially declared over on 16 January 2013, although all confirmed cases occurred in just a five day interval spanning 13-17 November.

Overall, 6 confirmed cases were reported with three confirmed deaths, giving a CFR of 50.0%.

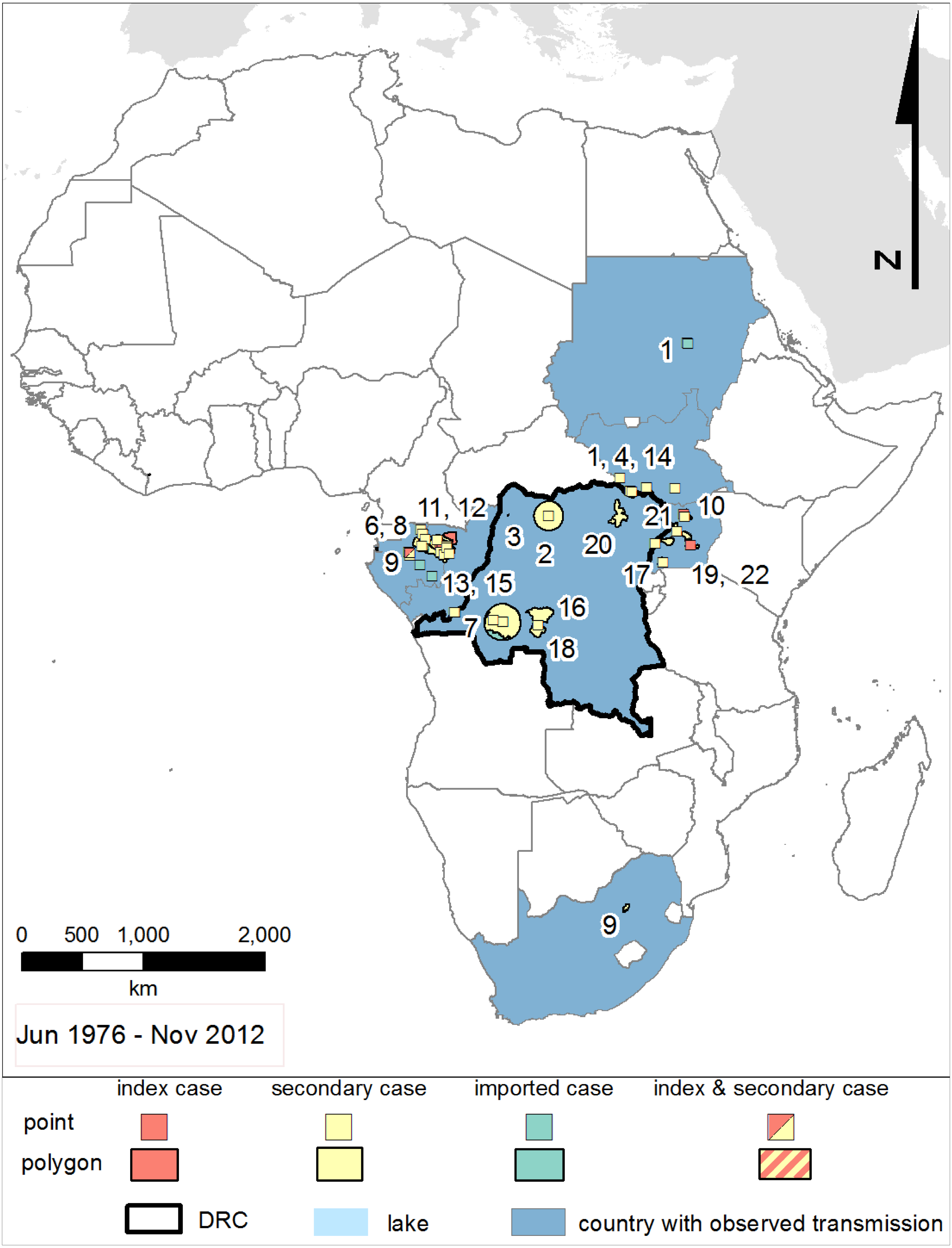

N
1
1, 4, 14
10
11, 12
21
6, 8
3
20
2
9
17
19, 22
13, 15
16
7
18
9
0 500 1,000 2,000
km
Jun 1976 - Nov 2012
index case
secondary case
imported case
index & secondary case
point
polygon
DRC
lake
country with observed transmission
